# A single dose investigational subunit vaccine for human use against Nipah virus and Hendra virus

**DOI:** 10.1038/s41541-021-00284-w

**Published:** 2021-02-08

**Authors:** Thomas W. Geisbert, Kathryn Bobb, Viktoriya Borisevich, Joan B. Geisbert, Krystle N. Agans, Robert W. Cross, Abhishek N. Prasad, Karla A. Fenton, Hao Yu, Timothy R. Fouts, Christopher C. Broder, Antony S. Dimitrov

**Affiliations:** 1 Galveston National Laboratory, University of Texas Medical Branch, Galveston, TX USA; 2 Department of Microbiology and Immunology, University of Texas Medical Branch, Galveston, TX USA; 3 Profectus BioSciences, Inc., Baltimore, MD USA; 4 Department of Microbiology and Immunology, Uniformed Services University, Bethesda, MD USA

**Keywords:** Protein vaccines, Viral infection

## Abstract

Nipah and Hendra viruses are highly pathogenic bat-borne paramyxoviruses recently included in the WHO Blueprint priority diseases list. A fully registered horse anti-Hendra virus subunit vaccine has been in use in Australia since 2012. Based on the same immunogen, the Hendra virus attachment glycoprotein ectodomain, a subunit vaccine formulation for use in people is now in a Phase I clinical trial. We report that a single dose vaccination regimen of this human vaccine formulation protects against otherwise lethal challenges of either Hendra or Nipah virus in a nonhuman primate model. The protection against the Nipah Bangladesh strain begins as soon as 7 days post immunization with low dose of 0.1 mg protein subunit. Our data suggest this human vaccine could be utilized as efficient emergency vaccine to disrupt potential spreading of Nipah disease in an outbreak setting.

## Introduction

Nipah virus (NiV) and Hendra virus (HeV) are enveloped, single-stranded negative-sense RNA viruses and the prototype members of the genus *Henipavirus* in the family Paramyxoviridae^1^. NiV and HeV are bat-borne disease-causing zoonoses, and *Pteropus* bats (flying foxes) are their major natural reservoir hosts although evidence in other bats exists^2^. In several animal species and in people, the major pathological observations of NiV and HeV infection is a severe systemic and often fatal neurologic and/or respiratory disease^3–5^. In addition, both NiV and HeV can also cause relapsing encephalitis, from several months to more than 10 years later, following a recovery from an acute infection as a result of recrudescence of virus replication in the central nervous system (CNS)^6,7^. NiV and HeV are also distinguished by their exceptionally broad species tropism and can cause disease in pigs, horses, cats, dogs, guinea pigs, hamsters, ferrets, squirrel monkeys, African green monkeys and humans^8^. NiV can also infect chicken embryos^9^. HeV was first recognized in 1994 as the cause of an outbreak that led to fatal cases of severe respiratory disease in horses and humans in the Brisbane suburb of Hendra, Australia. To date, HeV has appeared in Eastern Australia on 62 occasions causing the death or euthanasia of more than 100 horses, two HeV antibody-positive and euthanized dogs, and 4 fatalities of 7 human cases^10,11^. The horse is the predominant target host acquiring HeV infection from virus-shedding bats and is also the source of human infection^12^. NiV was later recognized as the agent causing a large outbreak of encephalitis among pig farmers in Malaysia in 1998/1999. Although NiV from Malaysia (NiV-M) has not re-emerged in Malaysia, nearly annual outbreaks of human cases of NiV infection have been recorded since 2001 in Bangladesh and a few in India caused by NiV-Bangladesh (NiV-B)^10^. The outbreaks of NiV-B have had notably higher human fatality rates ranging from 75-100%, along with the direct food-borne transmission of NiV from bats to humans and significant human-to-human transmission of NiV infection^13^. Evidence of NiV has also been detected in cattle, goats, and pigs^14^. However, in 2014, an outbreak of NiV encephalitis (attributed to the NiV-Malaysia strain) occurred in the Philippines with 9 fatalities of 11 human encephalitic cases and an influenza-like illness in another 6 individuals, one also with meningitis^15^. The Philippines outbreak involved horse-to-human and human-to-human transmission of NiV infection as well as apparent lethal infections of both cats and dogs. A more recent 2018 NiV-B outbreak in Kerala, India, occurred in a new geographic region far from the locations in Bangladesh and India where all prior appearances have occurred and had a high case fatality—91%, with 22 of 23 cases the result of human-to-human infection transmission at three different hospital locations^16^. Together, there have been over 650 cases of human NiV infection with a combined ∼60% case fatality rate, in South Asia and Southeast Asia across five countries^17^.

NiV and HeV are classified biological safety level 4 (BSL-4) viruses listed in Category C biothreat agents by the NIH and CDC. Nipah virus and henipaviral disease, which includes Hendra virus and possibly other viruses within the genus^18^ are included in the 2018 annual review of the WHO Blueprint list of priority diseases (https://www.who.int/blueprint/priority-diseases/en/). These emerging henipaviruses, unlike other Category A biothreat viral agents such as Smallpox virus or Zaire ebolavirus, can be isolated from natural reservoirs, grown in cell culture to high titers^1,9,19^, and spread in livestock, which is proven virus-amplifying sources for transmission to humans, where subsequent human-to-human aerosol transmission (NiV) can occur^20^. These factors make NiV and HeV important transboundary biological threats^10^. Indeed, NiV has the potential to be a pandemic threat since it can infect humans directly from natural reservoirs (bats) or indirectly following amplification in a susceptible animal species (livestock) and has a recognized capacity of nosocomial, human-to-human, and corpse-to-human transmission^21–23^. Currently, there are no approved human prophylaxis or therapeutic for NiV or HeV disease. A variety of therapeutic modalities have been explored as countermeasures to NiV and HeV infection with some assessed in animal challenge models, including ribavirin, chloroquine, polyIC12U, and heptad peptide fusion inhibitors but all with none to limited effectiveness^24^. Whereas vaccination, likely offers a viable anti-henipavirus strategy^25^ that could provide a significant public health benefit in endemic areas as well as provide an important component of countermeasure strategies to mitigate an outbreak of these viruses naturally or by deliberate means. To date, the most extensively studied NiV/HeV vaccine has been the soluble recombinant G glycoprotein of HeV (HeV-sG)^10^. The recombinant HeV-sG subunit glycoprotein is an inherently safe and highly effective vaccine that has been demonstrated to provide complete protection against lethal challenges of either NiV-M, NiV-B, or HeV in four animal species (cats, ferrets, nonhuman primates, and horses)^26–32^. The effectiveness of the HeV-sG immunogen led to the development and release of the equine anti-HeV vaccine (Equivac^®^ HeV) marketed by Zoetis, Inc., in Australia in 2012 under the Australian Pesticides and Veterinary Medicines Authority (APVMA) and is the first commercialized vaccine against any BLS-4 agent. The APVMA granted full registration of Equivac^®^ HeV in August 2015.

The HeV-sG recombinant antigen as subunit vaccine for use in people against Nipah and Hendra virus infection and disease has been in preclinical development since 2012 and it is currently in clinical development as an emergency vaccine countermeasure for Nipah virus outbreaks. Here, we report significant advancements in demonstrating the efficacy of a NiV/HeV human vaccine formulation in the African green monkey (AGM) model. The AGM model has been shown to properly represent NiV and HeV disease as reported in humans^33,34^. Thus, the efficacy of the HeV-sG subunit vaccine formulated for use in humans can be reliably tested in AGMs in the absence of any clinical trial evaluation in endemic areas of NiV disease. Here, we report results from two AGM challenge studies using the HeV-sG subunit vaccine. The first study (Study 1) showed that single-dose immunization was as efficient as the standard prime/boost regimen that has been tested in all previous studies. The second study (Study 2) showed that single-dose immunization protected AGMs from lethal NiV challenge as early as 7 days post-immunization. Together, these results: (i) single-dose immunization is protective; and ii) the protection starts as early as 1-week post-immunization; now provide evidence that the HeV-sG subunit vaccine formulation, for use in humans, is potentially suitable as an emergency vaccine countermeasure against outbreaks of NiV infection and disease, and also one that adheres to the target product profile for a safe and effective emergency-use NiV vaccine put forth by the WHO in 2017 (https://www.who.int/blueprint/priority-diseases/key-action/Nipah_virus_vaccineTPP.pdf?ua=1).

## Results

### Study 1—single-dose HeV-sG vaccine (HeV-sG-V) protects AGMs from either NiV or HeV infection after lethal challenges

**Fig. 1:**
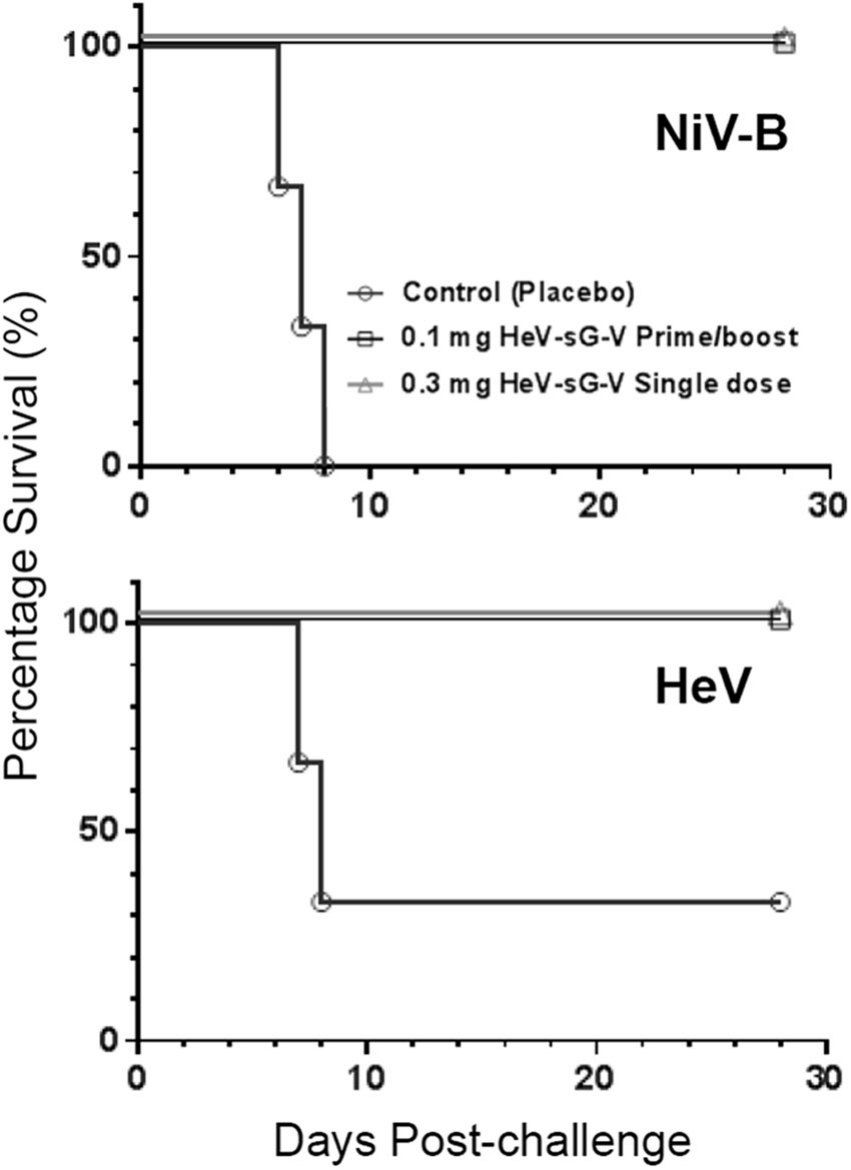
Kaplan–Meier plots—survival of the AGMs immunized with HeV-sG-V versus the placebo after challenge with NiV-B or HeV. The symbols for all subjects from both immunized groups, single-dose noted with triangles, ∆, and prime/boost regimen noted with squares, □, overlap on the plot. Each of the three non-immunized controls for each challenge is noted with a circle, ○.

The study design is summarized in Table 1 and described in the method section. Kaplan–Meier plots showing the survival of the subjects treated with HeV-sG-V versus the placebo after challenge with either HeV or NiV-B are shown in Fig. 1. Within 6–8 days post challenge, all subjects from the control group showed classical clinical signs of Henipavirus infection and disease, including depression, loss of appetite, lethargy, increased abdominal and labored breathing. Animals that developed acute respiratory distress and became unresponsive to the handler’s approach were humanely euthanized. From the control group, all three subjects challenged with NiV succumbed to the infection. Two of the three subjects from the HeV-challenged control group succumbed to the infection. The third subject, male, showed hallmark symptoms of infection, including depression, loss of appetite, and increased abdominal breathing, it recovered and survived until the endpoint of the study. In contrast to the controls, none of the HeV-sG-V vaccinated subjects showed clinical signs of illness and all of them survived until the study endpoint. Symptoms for clinical illness and clinical pathology records are detailed in Supplementary Table. 1.

**Table 1:** Design of the HeV-sG vaccine (HeV-sG-V) efficacy study 1.

Group	Treatment	Prime (Day 0)		Boost (Day 28)		
		Dose (mg)	Volume (mL)	Dose (mg)	Volume (mL)	Challenge virus^a^ (Day 56)
Control (*n* = 6)	Alhydrogel^b^	0	1	0	1	HeV (*n* = 3) NiV (*n* = 3)
Single dose (*n* = 6)	HeV-sG-V^c^	0.3	3^d^	None	n/a	HeV (*n* = 3) NiV (*n* = 3)
Prime/boost (*n* = 12)	HeV-sG-V^c^	0.1	1	0.1	1	HeV (*n* = 6) NiV (*n* = 6)

^a^The challenge virus was delivered intratracheally.

^b^Control was Alhydrogel diluted to contain 1.0 mg/mL Al^3+^ aluminum hydroxide suspension.

^c^HeV-sG-V contains 0.1 mg/mL HeV-sG in 1.0 mg/mL Al^3+^ aluminum hydroxide suspension.

^d^The 3.0 mL dosing volume was administered as 3 × 1 mL injections, intramuscularly to lumbar and thighs bilaterally. The 1 mL dose was delivered as a single injection.

Total 6 groups were created from 24 AGMs—3 duplet groups, HeV or NiV-B challenge group in each duplet. The two control groups (3 subjects per group) received a placebo on days 0 and 28. The two single-dose groups (3 subjects per group) received 0.3 mg HeV-sG in 3.0 ml HeV-sG-V on day 0. The two prime/boost groups (6 subjects per group) received 0.1 mg HeV-sG in 1.0 ml HeV-sG-V on day 0 and the same quantities repeated on day 28. All subjects were challenged on day 56 with the respective HeV or NiV-B. Animals were studies for another 28 days and euthanized on day 84.

**Fig. 2:**
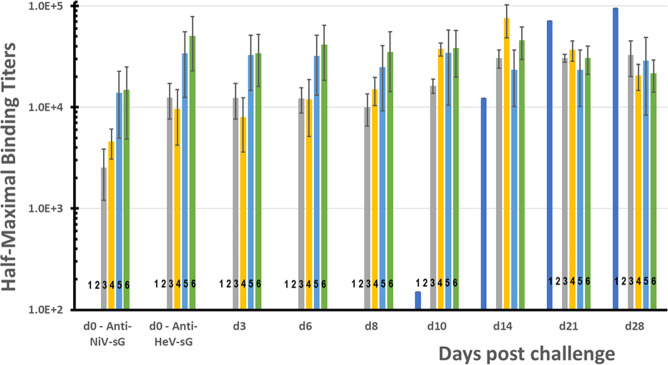
Post-challenge anti-HeV-sG antibody binding titers clustered by the day of sera sampling. The first cluster from the left shows titers for binding to NiV-sG antigen on the day of the challenge, d0. All other clusters show titers for binding to vaccine subunit HeV-sG antigen at the day of the challenge, d0, and at days 3, 6, 8, 10, 14, 21, and 28 post challenge. The bars in each cluster, numbered from 1 to 6, show the average titer and the standard deviation for sera samples from Study 1 AGM groups: bars 1—control group in HeV challenge (these bars show the titers for the single survived AGM-1-C1-2); bars 2—control group in NiV challenge; bars 3—single-dose group in HeV challenge; bars 4—single-dose group in NiV challenge; bars 5—prime/boost group in HeV challenge; and bars 6—prime/boost group in NiV challenge. Error bars show standard deviations.

Overall, HeV-sG-V elicited a robust humoral response among vaccinated subjects with serum neutralizing antibodies reactive to both HeV and NiV. Immunized animals were protected and demonstrated no clinical symptoms of the diseases. A plotted summary of the individual sera samples anti-HeV-sG antibody titers ELISA results are shown in Fig. 2. The ELISA data are represented as half-maximum antibody binding titers with units of measure in “ELISA units” (EU). On the day of the challenge, the control group tested negative for anti-HeV-sG antibodies (titers < 100 EU). By contrast, all of the HeV-sG-V-dosed animals had anti-HeV-sG antibody titers typical of a vaccine-induced immunological response. Titers were lower for the single-dose subject on the day of the challenge, however, until day 14 post challenge all immunized subjects, also the surviving HeV-challenged control, had similarly high anti-HeV-sG antibody titers. The single surviving male infected with HeV virus had a detectable binding titer of 151 EU by day 10, which rapidly increased toward the study endpoint. The serum samples from the day of challenge in this study (study D56) were also analyzed for antibodies to NiV soluble G protein (NiV-sG)—see Fig. 2.

**Fig. 3:**
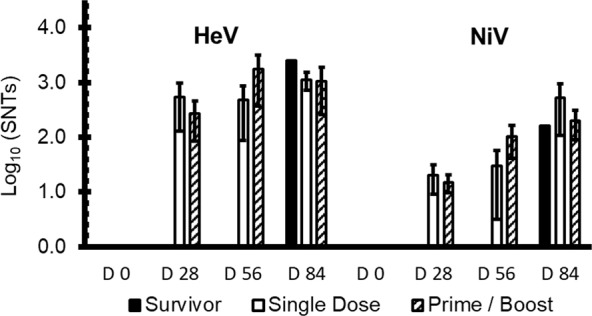
Average serum neutralization titers (SNTs) against either HeV or NiV-B infection in live virus neutralization plaque assay. Left 4 bar clusters show SNTs against HeV. Right 4 bar clusters show SNTs against NiV-B. Bars are clustered by the day of the study, where D0 is the immunization day, D28 is the day of the boost, D56 is the day of challenge, and D84—study end. White bars show the SNTs for single-dose immunized subjects, shaded bars—for the prime/boost immunized subjects, and the solid black bars—for the single survived control in the HeV challenge, AGM-1-C1-2. Error bars show standard deviations.

Sera neutralization titers (SNT) against either of HeV or NiV-B infection were tested for samples on the days of vaccinations, D0 and D28, day of the challenge, D56, and the day of study termination, D84. The titers to inhibit HeV infection (320–2560) were on average an order higher than the titers to inhibit NiV-B infections (10–160) on the challenge D56. SNTs against HeV and NiV-B were virtually in the same range (80–640) on the study termination D84. The HeV control that survived the challenge developed a serum with high neutralization titers on D84, 2560 against HeV and 160 against NiV-B infections, which is in line of titers developed by immunized subjects. Log10 of the average titer results are charted in Fig. 3. Results show that there is a tendency for the D28 neutralization titers to be higher for the single-dose vaccination groups, indicating dose dependence of the immune response. The SNTs on D56 is higher for the prime/boost vaccination group due to the boost immunization on D28. However, there is no sufficient statistics to claim the obvious difference in the SNTs resulting from the single-dose or prime/boost immunization schedule.

No viremia could be detected in vaccinated animals, while controls were severely viremic.

Viral RNA was not detected in the plasma of any of the AGMs vaccinated with HeV-sG-V. By contrast, all the AGMs from the placebo control group had detectable plasma viral RNA within 3–6 days post challenge (Supplementary Fig. 1A). The levels of viral RNA in the animals that succumbed to infection ranged from 3.49E + 07 to 6.98E + 8 copies/mL on the day of euthanasia. The single surviving male from the control group (challenged with HeV) had a significantly lower peak plasma viremia of 9.18E + 06 copies/mL on day 8 post challenge which declined below the level of detection by the end of the study (Supplementary Fig. 1B).

HeV, or respectively, NiV RNA was detected in all the tissues tested at levels from 1.15 × 10^6^ to 3.52 × 10^11^ (average 1.47 × 10^10^) copies for the control group AGMs that succumbed to infection. The single surviving male from the control group (challenged with HeV) had tissue viremia that was 3–5 logs lower than the non-surviving AGMs or, in some cases, below the level of detection (Supplementary Fig. 2). Among vaccinated subjects, 17 of the 18 AGMs had no detectable viral RNA in the tissues tested. AGM-1-3-3 (female) from the 0.1 mg prime/boost group had viral RNA in the lung, spleen, adrenal gland, axillary and inguinal LNs, heart, pancreas, and urinary bladder. Tissue viremia for AGM-1-3-3 was 3–5 logs lower than the non-surviving AGMs from the control group. Viral RNA was below the limit of detection (LOD) in the kidney and liver.

**Fig. 4:**
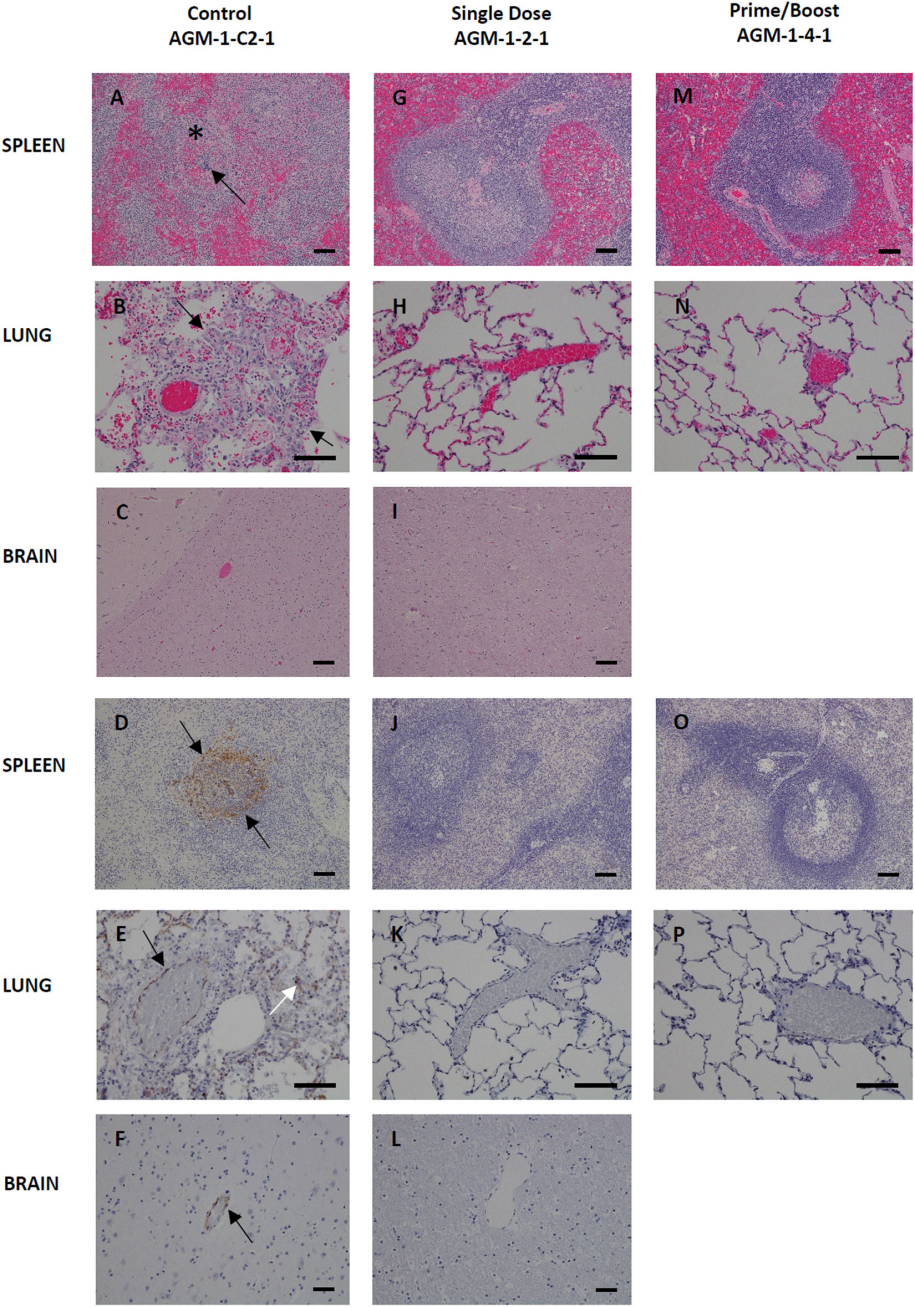
Histopathology and Immunohistochemistry (IHC) of NiV infected AGM tissues. Hematoxylin and Eosin (H&E) staining of representative tissues (**A**–**I**, **M**, and **N**) and NiV antigen staining for IHC images are indicated in brown (**D**–**F**, **J**–**L**, **O**, and **P**). Positive-control AGM for NiV (Control AGM-1-C2-1) includes images **A**–**F**. Splenic lymphoid necrosis (*) with syncytial cell formation (arrow) (**A**) with associated diffuse cytoplasmic immunolabeling of mononuclear cells (arrows) within the white and red pulp (**D**) interstitial pneumonia with alveolar hemorrhage, fibrin, foamy alveolar macrophages and cellular debris (arrows) (**B**) with associated immunolabeling of endothelium (black arrow), alveolar septate (white arrow) and alveolar macrophages (**E**), diffuse gliosis (**C**) with associated immunolabeling of the endothelium of small caliber vessels (arrow) within the brain (**F**). No lesions or immunolabeling was noted in representative tissues of Single dose of vaccine (single dose AGM-1-2-1) images **G**–**L** and prime with a boost of vaccine (Prime/Boost AGM-1-4-1) images **M**–**P**. H&E and IHC images of the spleen and brain were captured at 10× and lung at 20× magnification. Error bars—100 µm.

**Fig. 5:**
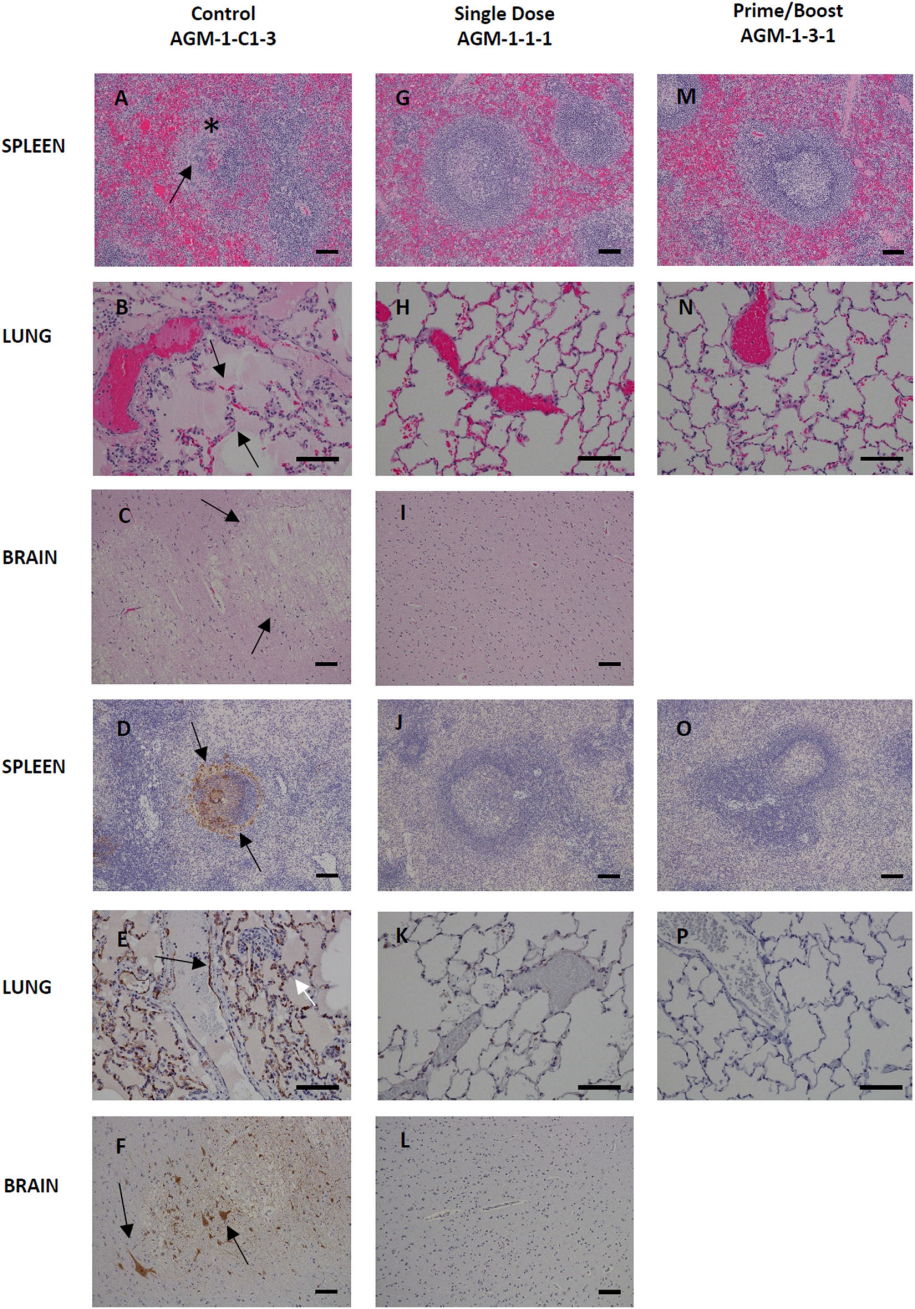
Histopathology and Immunohistochemistry (IHC) of HeV infected AGM tissues. Hematoxylin and Eosin (H&E) staining of representative tissues (**A**–**C**, **G**, **H**, **I**, **M**, and **N**) and HeV antigen staining for IHC images are indicated in brown (**D**–**F**, **J**–**L**, **O**, and **P**). Positive-control AGM for HeV (Control AGM-1-C1-3) includes images **A**–**F**. Splenic lymphoid necrosis (*) with syncytial cell formation (arrow) (**A**) with associated diffuse cytoplasmic immunolabeling of mononuclear cells within the white and red pulp (arrows) (**D**) interstitial pneumonia (arrows) with alveolar hemorrhage, fibrin, foamy alveolar macrophages, and cellular debris (**B**) with associated immunolabeling of endothelium (black arrow), alveolar septate (white arrow) and alveolar macrophages (**E**), diffuse gliosis with vacuolar plaque (arrows) (**C**) with associated immunolabeling of neuronal cells (arrows) and endothelium of small caliber vessels within the brain (**F**). No lesions or immunolabeling was noted in representative tissues of Single dose of vaccine (Single dose AGM-1-1-1) images **G**–**L** and prime with a boost of vaccine (Prime/Boost AGM-1-3-1) images **M**–**P**. H&E and IHC images of the spleen and brain were captured at 10× and lung at 20× magnification. Error bars—100 µm.

The necropsy data showed the diseases affected LNs, spleen, adrenal gland, lung, brain, cervical spinal cord, pituitary gland—Figs. 4 and 5. These organs were preserved in the immunized subjects or occasionally there was a very minor influence on the lung, spleen, liver, or kidneys. NiV-infected AGMs (AGM-1-C2-1, AGM-1-C2-2, and AGM-1-C2-3) displayed histologic lesions consistent with Nipah infection. Significant lesions included: moderate interstitial pneumonia and alveolar hemorrhage, fibrin and edema with endothelial syncytial cell formation, moderate lymphoid necrosis with syncytial cell formation of the splenic white pulp, and diffuse gliosis of the brain—Fig. 4 subsets A–F. Interstitial pneumonia is characterized by diffuse thickening of alveolar septae by moderate numbers of lymphocytes, plasma cells, histiocytes and fewer numbers of granulocytes, polymerized fibrin, and edema fluid. The alveolar spaces are flooded by edema fluid, polymerized fibrin, foamy alveolar macrophages, and cellular debris. Endothelial syncytial cells are most apparent in medium to small-caliber vascular spaces. Strong immunoreactivity for Nipah viral antigen was present within the endothelium (including syncytial cells), alveolar septae, and scattered alveolar macrophages—Fig. 4 subsets B and E. Multifocal follicular germinal centers in the splenic white pulp are moderately depleted of lymphoid elements and are effaced by hemorrhage, fibrin, karyorrhectic cellular debris, and syncytial cell formation. Strong immunoreactivity for Nipah viral antigen was present within the endothelium, syncytial cells, and scattered mononuclear cells—Fig. 4 subsets A and D. The cerebrum, brainstem, and portions of the cerebellum displayed diffuse gliosis. Strong immunoreactivity for Nipah viral antigen was identified in scattered small-caliber vessels within the parenchyma brain (cerebrum, cerebellum, and brainstem) and within the meninges—Fig. 4 subsets C and F. NiV-challenged AGMs representing the single dose (AGM-1-2-1) and prime/boost (AGM-1-4-1) vaccinated groups failed to display histologic lesions consistent with Nipah infection—Fig. 4 subsets G–P.

HeV-infected AGMs (AGM-1-C1-3 and AGM-1-C1-1) displayed histologic lesions consistent with Hendra infection. Significant lesions included: moderate interstitial pneumonia and alveolar hemorrhage, fibrin and edema with endothelial syncytial cell formation, moderate lymphoid necrosis with syncytial cell formation of the splenic white pulp. Lesions within the brain included focal vacuolar plaque with necrosis, vasculitis, intracytoplasmic eosinophilic inclusions bodies within neurons, and diffuse gliosis—Fig. 5 subsets A–F. Interstitial pneumonia is characterized by diffuse thickening of alveolar septae by moderate numbers of lymphocytes, plasma cells, histiocytes, and fewer numbers of granulocytes, polymerized fibrin, and edema fluid. The alveolar spaces are flooded by edema fluid, polymerized fibrin, foamy alveolar macrophages, and cellular debris. Endothelial syncytial cells are most apparent in medium to small-caliber vascular spaces. Strong immunoreactivity for Hendra viral antigen was present within the endothelium (including syncytial cells), alveolar septae, and scattered alveolar macrophages—Fig. 5 subsets B and E. Multifocal follicular germinal centers in the splenic white pulp are moderately depleted of lymphoid elements and are effaced by hemorrhage, fibrin, karyorrhectic cellular debris, and syncytial cell formation. Strong immunoreactivity for Hendra viral antigen was present within the endothelium, syncytial cells, and scattered mononuclear cells—Fig. 5 subsets A and D. The cerebrum, brainstem, and portions of the cerebellum displayed diffuse gliosis. Strong immunoreactivity for Hendra viral antigen was identified in scattered small-caliber vessels within the parenchyma brain (cerebrum, cerebellum, brainstem, cervical spinal cord, and pituitary gland) and within the meninges. Occasionally focal clusters of neuronal cells in the mid to ventral pons region of the brainstem, the dorsal parietal lobe of the cerebrum in association with a vacuolar plaque, and the cerebrum medial to the hippocampus had stippled to diffuse cytoplasmic immunoreactivity. Rarely, focal clusters of granulocytes within the cerebellum had immunoreactivity—Fig. 5 subsets C and F.

HeV-positive control AGM (AGM-1-C1-2) that survived the virus challenge failed to display histologic lesions consistent with acute systemic HeV infection. However, diffuse gliosis was noted in the cerebrum, brainstem, and portions of the cerebellum. Occasionally, multifocal lymphoplasmacytic perivascular cuffing was noted in the mid temporal lobe of the cerebrum and in the brainstem, specifically in the dorsal portion of the medulla oblongata and in the mid-pons region with locally extensive marked gliosis and minimal parenchymal vacuoles. Diffuse cytoplasmic immunoreactivity for Hendra viral antigen was noted within neuronal cells associated with the inflammatory lesions described in the mid temporal lobe. All other inflammatory lesions failed to have associated immunoreactivity for Hendra viral antigen (images not shown). HeV-challenged AGMs representing the single dose (AGM-1-1-1) and prime/boost (AGM-1-3-1) vaccinated groups failed to display histologic lesions consistent with Hendra infection—Fig. 5 subsets G–P.

### Study 2—single-dose HeV-sG-V begins to protect seven days post-immunization

**Fig. 6:**
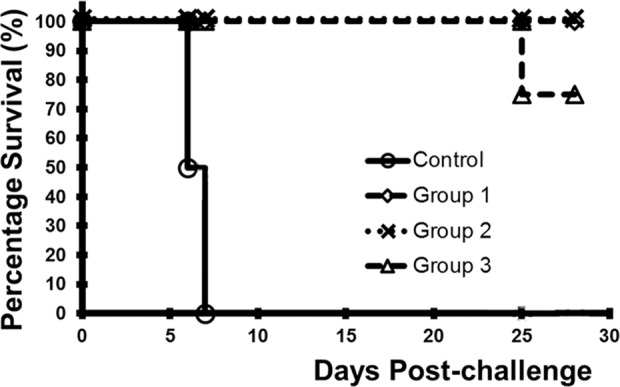
Kaplan–Meier plots showing survival of AGMs immunized with HeV-sG-V at 7 or 14 days before challenge with NiV-B. Description of the groups is given in Table 2. Symbols for all subjects from the immunized groups 1–3, noted with diamond, multiplication “×”, or triangle “∆”, respectively, overlap with one exception, AGM-2-3-1, which was abnormally lost on D25. The subjects from the control group are noted with circles, ο.

The study design is summarized in Table 2 and described in the method section. Table 3 shows individual clinical observations for the controls and two AGMs with disease symptoms following the NiV-B challenge. On days 6 and 7 post challenge, the control subjects showed clinical signs of NiV infection and disease, including depression, loss of appetite, lethargy, increased abdominal breathing, and/or labored breathing and succumbed to the infection. One of the subjects (male, AGM-2-1-3) in group 1 developed dyspnea and respiration quality issues on day 9 and recovered completely by day 12. The remaining 2 subjects from group 1 were healthy throughout the study with no disease symptoms. All four subjects in group 2 were healthy and free from visual symptoms until the study end, D28 when they were euthanized for further necropsy examinations. All 4 subjects from group 3 were free from disease symptoms until day 20, when one of them (female, AGM-2-3-1) developed weakness and mild depression with increasing scoring. On D22 it had moderate depression, hunched posture, and respiratory issues, and on D25 this subject was found immobile, not responsive, with decreased respiratory rate, and had to be euthanized according to protocol. Detailed clinical pathology and illness symptoms for all subjects in Study 2 are presented in Supplementary Table. 2. Kaplan–Meier surviving curves for the subjects treated with HeV-sG-V versus the placebo after the challenge with NiV-B are shown in Fig. 6.

**Table 2:** Design of the challenge protection Study 2.

Group	Treatment	Dose (mg)	Volume (mL)	Day of dosing	Challenge virus^a^
Control (*n* = 2)	Alhydrogel^b^	0	1	−7 one female −14 one male	NiV (D0)
Group 1 (*n* = 3)	HeV-sG-V^c^	0.1	1	−7	NiV (D0)
Group 2 (*n* = 4)	HeV-sG-V^c^	0.1	1	−14	NiV (D0)
Group 3 (*n* = 4)	HeV-sG-V^c^	0.3	3^d^	−14	NiV (D0)

^a^The challenge virus was delivered intratracheally.

^b^Control was Alhydrogel diluted to contain 1.0 mg/mL Al^3+^ aluminum hydroxide suspension.

^c^HeV-sG-V contains 0.1 mg/mL HeV-sG in 1.0 mg/mL Al^3+^ aluminum hydroxide suspension.

^d^The 3.0 mL dosing volume was administered as 3 × 1 mL injections, intramuscularly to lumbar and thighs bilaterally. The 1 mL dose was delivered as a single injection.

Four groups were created from 13 AGMs. The control group had two subjects. Group 1 of 3 subjects were immunized with 0.1 mg HeV-sG in 1.0 ml HeV-sG-V 7 days before the challenge. Group 2 of 4 subjects were immunized with the same vaccine dose 14 days before the challenge. Group 3 of 4 subjects were immunized with 0.3 mg HeV-sG in 3.0 ml HeV-sG-V 14 days before the challenge. We postulated the day of challenge to chosen as day 0 (D0) study day. Thus, the days of immunization were labeled with negative numbers: −7 for group 1 and −14 for groups 2 and 3. All subjects were euthanized on day 28.

**Table 3:** NiV-B disease clinical symptoms and death records for the individual AGMs that visually became infected after the NiV-B challenge in Study 2.

Observation	AGM-2-C-2	AGM-2-C-1	AGM-2-1-3	AGM-2-3-1
	Control	Control	Group 1	Group 3
Depression	Day 6 moderate/severe			Days 20–25 mild to severe
Weakness	Day 6			Days 20–25
Recumbency	Day 6			Day 25
Hunched posture	Day 6			Days 22–25
Dyspnea	Day 6	Day 7	Days 9–12	
Respiration	Day 6			Days 22 and 25
Death	Day 6	Day 8		Day 25

Typical symptoms that can usually be observed are lined down in the left column. The days the symptoms were actually observed are recorded for each symptomatic or succumbed to disease subject in the next columns. Detailed clinical pathology for all subjects in Study 2 are presented in Supplementary Table 2.

Both controls had recoverable NiV in the plasma and tissue samples, while none of the immunized subjects showed detectable viremia neither in blood nor in tissue samples. The subject from group 3, which was lost on day 25, was also free from viremia.

Viral RNA was detected in the plasma of the 3 vaccinated subjects from group 1 ranging from 5.2 to 6.7 log10 copies/ml between days 4 and 14 post-challenge (Supplementary Table 3). Viral RNA was cleared from the plasma of these animals and was below LOD since day 14 to the study end, day 28. No viral RNA was detected in the plasma samples from animals in groups 2 and 3 at any time point. By contrast, the subjects from the placebo control group had higher plasma viral RNA reading, above 7.0 log10 copies/ml day 7 post-challenge. The viral RNA from necropsy tissue of the control animals was detected in all of the tissues tested at levels from 5.45 to 9.74 log10 copies/ml (Supplementary Table 4). Most tissue samples had between 7 and 9 log10 copies/ml NiV RNA. By contrast, 7 of the 11 vaccinated animals had no detectable viral RNA in the tissues tested. Four vaccinated subjects had detectable viral RNA in some tissue samples: (i) one subject from group 1 had viral RNA in axillary LNs, inguinal LNs, spleen, kidney, and cervical spinal cord from 4.8 to 5.6 log10 copies/ml; (ii) another subject from group 1 had viral RNA in the spleen only, 5.8 log10 copies/ml; (iii) one subject from group 3 had detectable viral RNA in the liver, spleen, and brain stem, respectively with 5.3, 6.3, and 5.2 log10 copies/ml; and (iv) the subject from group 3, which was euthanized on day 25, had viral RNA in the brain stem and cervical spinal cord at 6.1 and 4.9 log10 copies/ml, respectively.

Sera neutralization titers were tested for samples on the day of vaccination, day of the challenge, and the day of termination. All SNTs tested for the vaccination day were as expected below LOD = 10. Results for the day of challenge and end of study day are presented in Supplementary Table 5. SNTs are below 10 on day 7 post-immunization, however, all subjects were protected from a lethal NiV-B challenge. SNTs elevated to an order of 10 on day 14 post immunization and raised to above 160 at the study end. There was no enough statistics to claim any difference between 0.1-mg and 0.3-mg dose groups. The group-3 subject that succumbed on day 25 had an SNT of 320 at the day of death, which is similar to the SNTs against NiV in Fig. 3.

## Discussion

There have been more than 10 NiV/HeV vaccine and challenge studies to date which have included several virus vectored strategies, virus-like-particles, mRNA, and recombinant protein subunits in a number of different animal models^10,17,25^. Among these, the HeV-sG recombinant protein subunit vaccine is the most extensively studied and evaluated vaccine against NiV and HeV to date. Further, the HeV-sG immunogen is also the most advanced in clinical development and is currently being evaluated in Phase I clinical trial sponsored by the Coalition for Epidemic Preparedness Innovations^35^ (CEPI). These efforts have focused on the HeV-sG subunit immunogen because of its proven cross-protective efficacy against NiV-M and NiV-B in at least three animal models including nonhuman primates, and because of the inherent safety of a recombinant subunit protein immunogen modality, and its effectiveness and success as a One-Health vaccine approach against the NiV/HeV transboundary viral threats^27^.

Presently, the ferret and AGM models are considered by the field to be the best representative animal models for replicating henipavirus infection and disease, as well as for the evaluation of potential anti-henipavirus therapeutics and vaccines. Previous studies by the authors along with others have extensively tested the HeV-sG recombinant subunit immunogen as a vaccine candidate in cats, ferrets, horses and AGMs. The original HeV-sG and NiV-sG immunization and NiV challenge experiments together with the development of the feline model demonstrated that HeV-sG was a superior immunogen and induced better cross-neutralizing antibody responses as compared to NiV-sG^32^. Those initial studies laid the groundwork demonstrating that a single sG immunogen (HeV-sG) can serve as vaccine against both HeV and NiV. These studies were followed by a series of additional immunization and challenge studies in the feline and ferret models^28,30,31^ followed by challenge studies in the horse against HeV and eventual licensure of HeV-sG as an equine vaccine^27^. The transition to the AGM model for further evaluation was critical, as it has been deemed highly probable that successful efficacy testing in a nonhuman primate model will likely be required for HeV-sG vaccine licensure^26,29^.

The results from the present studies now demonstrate that immunization with a single dose HeV-sG subunit human vaccine formulation induces a strong and completely protective immune response within a week post-immunization. This response eliminates the occurrence of severe disease and death in nonhuman primates after challenge with a lethal dose of either HeV or NiV-B in the same manner as previously described in prime-boost immunization modalities^33,34^.

In Study 1, the vaccine efficacy study, 3 groups of subjects were immunized with a single dose of 0.3 mg HeV-sG-V, and compare to the standard prime-boost immunization with a 0.1 mg HeV-sG-V vaccine dose, or a placebo consisting of the adjuvant/diluent as a control.

Here, these results confirm the protective immunogenic potency and now further substantiate the use HeV-sG-V as a prophylactic vaccine. A typical vaccination goal is the ability to elicit substantial immunity within one to two weeks following administration. A major question addressed in the present study was whether the HeV-sG-V could potentially serve as an emergency use vaccine and how soon post-immunization will protective immunity be efficient to prevent NiV infection and disease? In Study 2, we tested the effectiveness of HeV-sG-V as an emergency vaccination modality, whereby subjects immunized with a single 0.1-mg dose of HeV-sG-V were challenged with NiV at either 7- or 14-days post-vaccination. All three subjects in group 1 survived NiV challenge at just 7 days post-immunization. The fourth subject, which was pregnant, was excluded from the study because the increased immune tolerance is believed to be a major contributing factor to increased susceptibility and severity of infections during pregnancy^36^.

The circumstances underlying the loss of the single subject in Study 2 group 3 was uncertain. However, the blood chemistry data for this subject showed a steep elevation in the glucose, blood urea nitrogen, creatinine, amylase, and C-reactive protein after day 21 post-challenge reaching values of 314, 163, 5.1, 2590, and 100, respectively, at the day of death, day 25. Such high values were not reached by any other subjects in the study, including the controls at the day of death. Of note, the hematology results showed a decreased WBC count for this subject (1.8 × 10^3^ per µl), which was in contrast with the slight elevation in the WBC counts for infected subjects. Although it appeared this subject developed CNS clinical signs starting on day 20, this observation may not have been related to the virus challenge for the following reasons: (i) viral RNA was not detected in plasma; (ii) viral RNA was detected in 2 tissue samples; however, it was in line with occurrences of viral RNA in the other animals; (iii) viremia was not detected in any tissue sample; (iv) viremia was not detected in blood samples; and (v) the SNT titer on day 25 was 320, in line with that of the other immunized, and fully protected subjects. Together, these data strongly suggest that the underlying cause of the loss of the subject in group 3 was unrelated to the virus challenge.

The present findings demonstrate that a single dose of 0.1-mg HeV-sG, formulated for use in humans, was capable of inducing a completely protective immune response in nonhuman primates from a 5 × 10^5^ pfu challenge dose of NiV-B delivered by i.t. and i.n. administration at just 7 days post-vaccination. Here, because of the limitations to study size with NHPs in the BSL-4 setting, very high lethal NiV-B and HeV doses that cause uniform death in 6–8 days are required and were used in the present studies. However, it should be recognized that most naturally occurring infections of humans with NiV and HeV lead to case fatality rates ranging from 40 to 90% deaths with a disease course lasting a few to several weeks following infection. Thus, it may be likely that the effectiveness of the HeV-sG-V in preventing infection and disease may be substantially more efficacious under the setting of natural virus exposure and transmission conditions. In summary, these findings, together with the preponderance of other supportive data indicate that the HeV-sG-V is suitable for use as either an emergency or prophylactic vaccine for the prevention of infection and disease caused by NiV and HeV.

## Methods

### Hendra virus soluble G vaccine (HeV-sG-V) formulation for use in humans

The initial efficacy data were generated using HeV-sG in co-formulation with both Alhydrogel and CpG ODN 2006 adjuvants. To simplify human vaccine development, subsequent studies were performed using Alhydrogel™ only. Two studies in ferrets and one in AGM confirmed that HeV-sG formulated with Alhydrogel^™^ alone provides a fully protective vaccine at doses as low as 10 µg HeV-sG per dose. The ratio of HeV-sG /Alum in these studies, however, was kept at 1/25 as in the preliminary studies. Although 10 µg dose protected AGMs from NiV challenge, the immune responses were highly variable and one of the animals, with low immune response, showed a relatively high presence of viral RNA at the study end. Thus, 0.1 mg dose, which caused a more predictable and robust immune response was chosen as a tentative dose for use in humans. If a 0.1 mg dose HeV-sG of this formulation were to be tested in the clinic, it would contain 2.5 mg of Al^3+^, which is twice the maximum dose recommended by the FDA for single-site administration. Follow-on studies demonstrated that HeV-sG is fully absorbed to Alhydrogel^™^ at a ratio as low as 1/7. Therefore, a ratio of 1/10 HeV-sG /Alhydrogel^™^ provides 70% absorption saturation and a 0.1 mg dose of HeV-sG will contain 1 mg Al^3+^, which is 80% of the maximum allowed Al^3+^ dose per shot when Alhydrogel is used. A rabbit immunogenicity study demonstrated a 30 µg dose of HeV-sG formulated with Alhydrogel^™^ at ratios of 1/10 induced the same humoral response as at ratios of 1/25 (Supplementary Fig. 3). Thus, a 1/10 HeV-sG/Alhydrogel^™^ formulation for the HeV-sG-V was selected for development and clinical evaluation.

A HEK-293 Research Cell Bank (RCB) expressing HeV-sG protein (HeV-sG-HEK-293) was prepared by Profectus BioSciences, Baltimore, using multiple transductions of parent HEK-293F cells with a recombinant MoMuLV carrying the HeV-sG gene, followed by two consecutive rounds of limited dilution cloning. The productivity of the RCB was greater than 1 gm/L HeV-sG when grown in shake flasks using a preliminary optimized feeding schedule. The RCB was used as a seed to manufacture a HeV-sG-HEK-293 master cell bank (MCB) at Charles River Laboratories—lot#5407580MCB2, December 31, 2013, 325 vials, 1.16 × 10^7^ viable cells/vial. The MCB has passed all testing required by FDA and has been released by Profectus quality assurance.

Catalent Pharma, our CMO, has developed a scalable upstream process suitable for cGMP protein production, in which the concentration of HeV-sG in the supernatant exceeded 0.6 g/L, which was judged adequate for further development. A scalable downstream purification process suitable for cGMP manufacture of HeV-sG has also been developed. The process consists of: (1) Media clarification by depth filtration (3 M Zeta Plus 60SP02A); (2) Triton-X 100 added to 0.1% v/v and incubated for 1 h (for viral inactivation) followed by pH reduction to 5.0; (3) Capto MMC mixed-mode hydrophobic cation exchange chromatography with elution over a 20 column volume gradient starting with 100% 25 mM MES, pH 6.0 and moving to 25 mM MES, pH 6.0, 0.5 M NaCl followed by increase in pH to 8.0; (4) intermediate purification using CaptoAdhere mixed-mode hydrophobic anion exchange chromatography with elution over a 20 column volume gradient starting with 100% 50 mM Tris, pH 8.0 and moving to 50 mM Tris, pH 8.0, 1 M NaCl, followed by adding an equal volume 2 M ammonium sulphate, pH 7.5; 5) polishing purification using Phenyl HP hydrophobic interaction chromatography with elution over a 32 column volume gradient starting with 80% 50 mM Tris, 1 M ammonium sulfate, pH 7.5 and moving to 50 mM Tris, pH 7.5; 6) an additional polishing chromatography by diafiltration with 7 volumes 50 mM Tris, pH 7.5 followed by Sartobind Q membrane in flow-through mode with 50 mM Tris, pH 7.5; 7), and viral filtration by using a Virosart 150 CPV capsule filter. Finally, the HeV-sG solution was dialyzed against PBS (10 mM sodium phosphate, 150 mM NaCl, pH 7.0), concentrated to 9.84 mg/mL using tangential flow filtration with a 50 kD MWCO PES membrane, and sterile filtered through a 0.2-µm PES filter. This cGMP-like purification process was used to purify HeV-sG from the supernatant of a 10-L bioreactor run to obtain 2.5 g HeV-sG, 2×125 mL in PETG bottles at 9.84 mg/mL in PBS. This HeV-sG, called also a drug substance, was used to formulate the toxicology lot of HeV-sG/Alhydrogel^™^ vaccine. The toxicology lot of HeV-sG/Alhydrogel^™^ officially labeled as “0.1 mg/mL HeV-sG, 1.0 mg/mL Al^3+^ in Aluminium Hydroxide Suspension for Injection, (Toxicology Supplies), Lot PUP05401A” from 19 July 2016 was prepared at Catalent Pharma. 624 vials (each vial contains one dose vaccine) out of 649 totally filled were made available for stability testing, the GLP toxicology study, and animal efficacy testing studies that are reported here.

### Challenge viruses—Hendra virus (HeV) and Nipah virus (NiV)

*Hendra virus (HeV)* isolate of 1994 (Brisbane, Australia) was used in the vaccine efficacy study. The virus was isolated passed in Vero E6 cells. Vero p5 (Batch/Lot No. 80859, at ~1 × 10^7^ pfu/mL) was prepared by the University of Texas Medical Branch (UTMB) and utilized in this study. Vero E6 p1 stock (# 808593) was obtained from CDC, p2 stock was prepared in Vero E6 cells by Rocky Mountain Laboratories (RML; Hamilton, MT), Division of Intramural Research, National Institute of Allergy and Infectious Diseases, National Institutes of Health and transferred to University of Texas Medical Branch (UTMB). The virus is stored at −80 °C, tested negative for mycoplasma and endotoxin.

*Nipah virus (NiV)* Bangladesh isolate (NiV-B) # 200401066 from the human patient at Rajbari, Bangladesh, in 2004. P1 stock was obtained from CDC, p2 stock was prepared in Vero E6 cells and stored at University of Texas Medical Branch (UTMB) on 30 May 2011. The virus is stored at −80 °C, tested negative for mycoplasma and endotoxin.

### African green monkeys (AGM)

AGMs (*Chlorocebus aethiops*) from St. Kitts were used in both challenge studies. AGMs were purchased from PrimGen, Hines, IL. The vaccine efficacy study (Study 1) utilized 24 AGMs (12 male, 12 female) with body weights in the range of 3.4–5.7 kg (Supplementary Table 6) and the onset of protection against NiV challenge study (Study 2) utilized 14 AGMs (7 male, 7 female) with body weights in the range of 3.1–6.0 kg (Supplementary Table 7). Each animal was uniquely identified by means of a unique body tattoo.

Animal studies were performed at Galveston National Laboratory at the University of Texas Medical Branch (UTMB) under protocols approved by the UTMB Institutional Animal Care and Use Committee (IACUC). The UTMB facility is accredited by the Association for Assessment and Accreditation of Laboratory Animal Care, International (AAALAC).

#### Acclimation and housing

AGMs were received at UTMB 2 months prior to the start of the study. All animals passed a physical examination upon arrival and were certified as healthy. During the challenge phase of the study the animals were housed individually in the animal biosafety level 4 (ABSL-4) biocontainment in standard indoor primate housing. AGMs were provided with monkey chow and fruits daily, water ad libitum via an automatic watering system, and they were regularly provided with enrichment. All animals were checked at least once per day and animals that developed clinical signs were checked multiple times per day.

#### Anesthesia

Food was withheld the morning of all procedures requiring anesthesia. Because of the inherent risk in handling animals infected with a BSL-4 agent, AGMs were immobilized in their cages by the use of a squeeze device and sedated using an intramuscular injection with ketamine prior to phlebotomy, clinical examination, treatment, and/or challenge. A loss of motor reflexes and sensation to pain as evidenced by toe pinch and lack of movement was achieved before any procedures are performed on any animals.

#### Euthanasia

When the clinical observations and scores of the infected animals reached pre-defined levels from the UTMB IACUC protocols, AGMs were humanely euthanized under deep anesthesia in accordance with the American Veterinary Medical Association (AVMA) Guidelines on Euthanasia. At the study endpoint, all surviving AGMs were humanely euthanized for a final necropsy exam.

### Study 1 design—immunization and challenges with NiV or HeV, schedule

Vaccinations of the animals were performed under anesthesia. Male and female AGMs were divided equally across the groups. The vaccine doses were administered as intramuscular (i.m.) injections into the lumbar or thigh muscle(s). Vials were mixed to achieve a homogeneous suspension just prior to withdrawing the dose volume. The dose for the prime/boost group (*n* = 12) was 0.1 mg HeV-sG-V delivered as a single 1.0 mL injection on Days 0 and 28 (for a total of 2 dosing cycles) (Table 1). The single-dose group (*n* = 6) were vaccinated with 0.3 mg HeV-sG-V delivered as 3 separate 1.0 mL injections (bilateral lumbar and thigh) on Day 0 and did not receive any treatment on Day 28. The control group (*n* = 6) was dosed with 1.0 mL placebo on Days 0 and 28. On study day 56, animals were exposed to 5 × 10^5^ Pfu of either HeV (half of the animals in each group) or NiV (the other half in each group) with the dose being equally divided between the intratracheal (i.t.) and the intranasal (i.n.) routes. HeV isolate from Brisbane, Australia (1994) was used for the HeV challenge and NiV Bangladesh (NiV-B) isolate #200401066 from Rajbari, Bangladesh (2004) was used for the NiV challenge. The animals were observed for clinical signs of disease for 28 days until the study end on Day 84.

### Study 2 design—immunization and challenge schedule with NiV

Vaccinations of the animals were performed using the same protocol as in the previous efficacy study under anesthesia. The vaccine and the placebo were administered i.m. into the lumbar and/or thigh muscle(s). HeV-sG-V vials were mixed to achieve a homogeneous suspension just prior to withdrawing the dose volume. Male and female subjects were divided into four groups for the vaccine phase of the study (Table 2). The control group (*n* = 2) received a placebo (alhydrogel) on day −7 (one AGM) and day −14 (one AGM). Group 1 (*n* = 3) received 0.1-mg HeV-sG-V as a single 1.0 mL injection on day −7. Similarly, group 2 (*n* = 4) received 0.1-mg HeV-sG-V 1-mL injection on day 14. Group 3 received 0.3-mg HeV-sG-V on day −14. Dosing for group 3 was delivered as 3 separate 1.0 mL injections (bilateral lumbar and thigh). Initially, group 1 was designed to have 4 subjects; however, one of the subjects happened to be pregnant, thus, not meeting the inclusion criteria, and was excluded from further considerations. All subjects were immunized at ABSL-2 and transferred to ABSL-4 for the challenge phase of the study starting on D0. On D0, each animal was exposed to 5 × 10^5^ Pfu of NiV with the dose being equally divided between the i.t. and the i.n. routes. NiV-B isolate #200401066 from Rajbari, Bangladesh (2004) was used for this NiV challenge

### Clinical observations

Animals were given a physical exam upon arrival to the UTMB. During the challenge phase, detailed clinical observations were recorded daily from the day of entry into the ABSL-4 until study end including body temperature, weight, food consumption, respiration quality, activity levels, and neurological signs.

Cage-side observations were conducted on unanesthetized animals, whereas observations requiring manipulation (i.e., handling) of the animal were performed under anesthesia.

Body weights and temperatures measured rectally under anesthesia were recorded for each monkey during the challenge phase on: (i) 0, 3, 6, 8, 10, 14, 21, and 28 days post challenge in Study 1 and (ii) 0, 4, 7, 10, 14, 21, and 28 days post challenge in Study 2; or at the time of euthanasia for animals that succumbed to infection.

Blood specimens were collected from sedated animals (via the femoral artery) into serum separator tubes and tubes containing ethylenediaminetetraacetic acid (EDTA). Blood was collected on study days 0, 28, 56 (0), 59 (3), 62 (6), 64 (8), 66 (10), 70 (14), 77 (21), and 84 (28) post immunization (post challenge) or at the time of euthanasia for animals that succumbed to infection in Study 1. In Study 2 the sample collection days were −14, −7, 0, 4, 7, 10, 14, 21, and 28 post-challenge or at the time of euthanasia for animals that succumbed to infection. Supplementary Tables 8 and 9 show the assays performed on each blood draw.

Whole blood and serum were collected for hematology and serum biochemistry on study days (post-challenge days) 0, 28, 56 (0), 59 (3), 62 (6), 64 (8), 66 (10), 70 (14), 77 (21), and 84 (28) or at the time of euthanasia in Study 1 or study days −14, −7, 0, 4, 7, 10, 14, 21, and 28 post challenge or at the time of euthanasia in Study 2. The samples were assayed for changes in hematology and serum chemistry. Total white blood cell counts, white blood cell differentials, red blood cell counts, platelet counts, hematocrit values, total hemoglobin, mean cell volume, mean corpuscular volume, and mean corpuscular hemoglobin concentration were determined from blood samples collected in tubes containing EDTA using a laser-based hematologic analyzer (Beckman Coulter Brea, CA, USA). Serum samples were tested for concentrations of albumin, amylase, alanine aminotransferase, aspartate aminotransferase, alkaline phosphatase, gamma-glutamyltransferase (GGT), glucose, total protein, blood urea nitrogen, uric acid, calcium, C-reactive protein, and creatinine by using a Piccolo point-of-care blood analyzer and Biochemistry Panel Plus discs (Abaxis, Sunnyvale, CA, USA).

All animals were euthanized (when moribund or at the study endpoint on Day 28 post challenge) and were subjected to a complete necropsy examination by a veterinary pathologist, or another suitably qualified person with appropriate training and experience in animal anatomy and gross pathology. The exam included evaluation of the carcass and musculoskeletal system; all external surfaces and orifices; cranial cavity and external surfaces of the brain; and thoracic, abdominal, and pelvic cavities with their associated organs and tissues.

At the time of necropsy, tissue samples were collected for viral RNA analysis. Tissue samples were also collected and fixed in 10% formalin for histopathological and immunohistochemical analysis (data not included in this report).

### Binding titers for anti-HeV-sG or anti-NiV-sG antibodies

Testing for the presence of anti-HeV-sG antibodies was performed according to a characterized method (Profectus BioSciences SOP 339, “Quantification of Anti-HeV-sG Antibodies in Non-Human Primate Sera by ELISA”) in a nonregulated (non-GLP and non-GMP) discovery-based laboratory. A purified monoclonal anti-HeV-sG antibody, m102.4, that also binds to NiV-sG was included as a positive control with established acceptance criteria on each ELISA plate. In brief, each serum sample or control antibody was tittered onto 96-well plates coated with purified HeV-sG (or NiV-sG) protein. Anti-HeV-sG (or anti-NiV-sG) antibodies from the samples were captured on the plate and detected using a horseradish peroxidase (HRP)-conjugated goat anti-monkey IgG secondary antibody. TMB peroxidase substrate was added to produce a chromogenic reaction that was proportional to the amount of antibody on the plate. After stopping the reaction with acid, the 450 nm absorbance of each well was read using a SpectraMaxPlus 384 microplate spectrophotometer (Molecular Devices, LLC, Silicon Valley, California). Binding curves and half-maximum antibody binding titers were calculated using SoftMax Pro 5.4 microplate data analysis software (Molecular Devices).

### Sera neutralization titers (50% plaque reduction)

Sera neutralization titers were determined by microneutralization assay. Briefly, sera were heat-inactivated at 56 °C for 1 h, serially diluted two-fold, and incubated with 100 TCID_50_ of NiV for 1 h at 37 °C. Virus and antibodies were then added to a 96-well plate with 2 × 10^4^ Vero E6 per well in four wells per antibody dilution. Wells were checked for cytopathic effect (CPE) 3 days after infection, and the 50% neutralization titer was determined as the serum dilution, at which at least 50% of wells showed no CPE.

### Viral RNA detection from blood and tissue samples

Whole blood was collected for viral RNA analysis during the challenge phase on 0, 3, 6, 8, 10, 14, 21, and 28 days post challenge or at the time of euthanasia. RNA was extracted from 100 µl whole blood utilizing 600 µl of AVL viral lysis buffer and the QIAamp Viral RNA Mini Kit (QIAGEN).

At the time of necropsy, tissues were sampled for viral RNA including axillary lymph node (LN), inguinal LN, liver (with gallbladder), spleen, kidney, adrenal gland, right lung upper lobe, right lung middle lobe, right lung lower lobe, left lung upper lobe, left lung middle lobe, left lung lower lobe, brain frontal lobe, brain stem, cervical spinal cord, mandibular LN, submandibular saliva gland, tonsil, heart, mesenteric LN, duodenum, pancreas, ileocecal junction and cecum, transverse colon, urinary bladder, gonad (testis or ovary), uterus or prostate, nasal mucosa, conjunctiva, and eye. Tissue samples were stabilized in RNAlater storage buffer. Approximately, 100 mg of each tissue sample was homogenized in 600 µl of RLT lysis buffer and RNA was extracted using the RNeasy Mini Kit (QIAGEN).

RNA isolated from blood and tissues was analyzed with strain-specific primers/probes targeting the nucleocapsid (N) gene for quantitative real-time PCR (qRT-PCR). Primers were designed to target the intergenic region, which allowed for genome and anti-genome detection only without detecting contaminating viral mRNA (NiV-B primers: Fwd 5′-CAA GAT CTC AAA CCC ACT CAA-3′, Rev 5′-TAA TGT AAT TGG TCC CTT AGT GTT G-3′; and HeV primers: Fwd 5′-CAT CGG AAA GAA ACC CAC CTA A-3′, Rev 5′-GGT TTG GGT TCC TGG TCA TAT-3′). All probes were obtained from Life Technologies, Carlsbad, CA. Viral RNA was detected using the CFX96 detection system (Bio-Rad Laboratories, Hercules, CA) with One-Step Probe qRT-PCR kits (QIAGEN). Threshold cycle (CT) values representing viral genomes were analyzed with CFX Manager Software, and data are shown as genome equivalents (GEq)/ml. To create the GEq standard, RNA from viral stocks was extracted and the number of strain-specific genomes was calculated using Avogadro’s number and the molecular weight of each viral genome.

### Infectious virus detection from blood and tissue samples

Virus titration was performed by plaque assay with Vero cells from all plasma and tissue (10% homogenate) samples. Briefly, increasing 10-fold dilutions of the samples were adsorbed to Vero cell monolayers in duplicate wells (200 μl); the LOD was 25 pfu/ml for plasma and 250 pfu/gram for tissues.

### Histopathology and immunochemistry

The following tissues were collected at necropsy and evaluated histologically: spleen, lung, and brain tissues from the frontal cortex of the cerebrum, cerebellum, hippocampus, brainstem, cervical spinal cord, and pituitary gland.

Tissues were fixed in 10% neutral buffered formalin, for a minimum of 21 days. The formalin solution was replaced once. Tissues were placed in cassettes and processed with a Shandon Excelsior, on a 12-h automated schedule, using a graded series of ethanol, xylene, and ParaPlast Extra. Embedded tissues are sectioned at 5 µm and dried overnight at 60 °C prior to staining.

Specific anti-Nipah immunoreactivity was detected using an anti-Nipah N protein rabbit 345 primary antibodies (gift from Dr. Linfa Wang to Dr. Broder) at a 1:4000 dilution or 30 min. The tissues were processed for immunohistochemistry using the Dako Autostainer. The secondary antibody was biotinylated goat anti-rabbit IgG (Vector Labs #BA-1000) used at 1:200 dilution for 30 min followed by Dako LSAB2 streptavidin-HRP (#K1016) for 15 min. Slides were developed with Dako DAB chromagen (#K3468) for 5 min and counterstained with Harris Hematoxylin for 1 min.

### Reporting summary

Further information on experimental design is available in the Nature Research Reporting Summary linked to this paper.

## Supplementary information

Supplementary Information

Reporting Summary

## Data Availability

All substantial data is available in the text and the Supplementary Figures and Tables. Certificates of analysis and origins, material data sheets, and detailed procedures are available upon request to the corresponding author. Materials generated will be available after the appropriate material transfer agreement.

## References

[1] Wang, L.-F., Mackenzie, J. S. & Broder, C. C. in *Fields Virology* Vol. 1 (eds D. M. Knipe & P. M. Howley) Ch. 37, 1070–1085 (Lippincott Williams & Wilkins, 2013).

[2] Clayton BA, Wang LF, Marsh GA (2013). Henipaviruses: an updated review focusing on the pteropid reservoir and features of transmission. Zoonoses Public Health.

[3] Abdullah S, Tan CT (2014). Henipavirus encephalitis. Handb. Clin. Neurol..

[4] Wong KT, Ong KC (2011). Pathology of acute henipavirus infection in humans and animals. Pathol. Res. Int..

[5] Playford EG (2010). Human Hendra virus encephalitis associated with equine outbreak, Australia, 2008. Emerg. Infect. Dis..

[6] Wong, K. T. & Tan, C. T. Clinical and pathological manifestations of human henipavirus infection. *Curr. Top. Microbiol. Immunol.*10.1007/82_2012_205 (2012). 10.1007/82_2012_205 22427144

[7] Wong KT (2009). Human Hendra virus infection causes acute and relapsing encephalitis. Neuropathol. Appl. Neurobiol..

[8] Geisbert TW, Feldmann H, Broder CC (2012). Animal challenge models of henipavirus infection and pathogenesis. Curr. Top. Microbiol. Immunol..

[9] Tanimura N, Imada T, Kashiwazaki Y, Sharifah SH (2006). Distribution of viral antigens and development of lesions in chicken embryos inoculated with nipah virus. J. Comp. Pathol..

[10] Broder CC, Weir DL, Reid PA (2016). Hendra virus and Nipah virus animal vaccines. Vaccine.

[11] Government, Q. *Summary of Hendra Virus Incidents in Horses*. https://www.business.qld.gov.au/industries/service-industries-professionals/service-industries/veterinary-surgeons/guidelines-hendra/incident-summary (2019).

[12] Field HE (2016). Hendra virus ecology and transmission. Curr. Opin. Virol..

[13] Chattu VK, Kumar R, Kumary S, Kajal F, David JK (2018). Nipah virus epidemic in southern India and emphasizing “One Health” approach to ensure global health security. J. Fam. Med. Prim. Care.

[14] Chowdhury S (2014). Serological evidence of henipavirus exposure in cattle, goats and pigs in Bangladesh. PLoS Negl. Trop. Dis..

[15] Ching PK (2015). Outbreak of henipavirus infection, Philippines, 2014. Emerg. Infect. Dis..

[16] Arunkumar G (2019). Outbreak Investigation of Nipah Virus Disease in Kerala, India, 2018. J. Infect. Dis..

[17] Amaya M, Broder CC (2020). Vaccines to emerging viruses: Nipah and Hendra. Annu. Rev. Virol..

[18] Thibault PA, Watkinson RE, Moreira-Soto A, Drexler JF, Lee B (2017). Zoonotic potential of emerging paramyxoviruses: knowns and unknowns. Adv. Virus Res..

[19] Crameri G, Wang LF, Morrissy C, White J, Eaton BT (2002). A rapid immune plaque assay for the detection of Hendra and Nipah viruses and anti-virus antibodies. J. Virol. Methods.

[20] Luby SP, Gurley ES (2012). Epidemiology of henipavirus disease in humans. Curr. Top. Microbiol. Immunol..

[21] Luby SP (2013). The pandemic potential of Nipah virus. Antivir. Res..

[22] Chakraborty A (2016). Evolving epidemiology of Nipah virus infection in Bangladesh: evidence from outbreaks during 2010–2011. Epidemiol. Infect..

[23] Sazzad HM (2013). Nipah virus infection outbreak with nosocomial and corpse-to-human transmission, Bangladesh. Emerg. Infect. Dis..

[24] Broder CC (2012). Henipavirus outbreaks to antivirals: the current status of potential therapeutics. Curr. Opin. Virol..

[25] Broder CC (2012). Immunization strategies against henipaviruses. Curr. Top. Microbiol. Immunol..

[26] Mire CE (2014). A recombinant Hendra virus G glycoprotein subunit vaccine protects nonhuman primates against Hendra virus challenge. J. Virol..

[27] Middleton D (2014). Hendra virus vaccine, a one health approach to protecting horse, human, and environmental health. Emerg. Infect. Dis..

[28] Pallister JA (2013). Vaccination of ferrets with a recombinant G glycoprotein subunit vaccine provides protection against Nipah virus disease for over 12 months. Virol. J..

[29] Bossart KN (2012). A Hendra virus G glycoprotein subunit vaccine protects African green monkeys from Nipah virus challenge. Sci. Transl. Med..

[30] Pallister J (2011). A recombinant Hendra virus G glycoprotein-based subunit vaccine protects ferrets from lethal Hendra virus challenge. Vaccine.

[31] McEachern JA (2008). A recombinant subunit vaccine formulation protects against lethal Nipah virus challenge in cats. Vaccine.

[32] Mungall BA (2006). Feline model of acute nipah virus infection and protection with a soluble glycoprotein-based subunit vaccine. J. Virol..

[33] Geisbert TW (2010). Development of an acute and highly pathogenic nonhuman primate model of Nipah virus infection. PLoS ONE.

[34] Rockx B (2010). A novel model of lethal Hendra virus infection in African green monkeys and the effectiveness of ribavirin treatment. J. Virol..

[35] Gouglas D, Christodoulou M, Plotkin SA, Hatchett R (2019). CEPI: driving progress toward epidemic preparedness and response. Epidemiol. Rev..

[36] Kourtis AP, Read JS, Jamieson DJ (2014). Pregnancy and infection. N. Engl. J. Med.

