# Supplementary material for: A single dose investigational subunit vaccine for human use against Nipah virus and Hendra virus
Source: NPJ Vaccines. 2021 Feb 8;6:23. doi: 10.1038/s41541-021-00284-w (PMC7870971; doi:10.1038/s41541-021-00284-w)
Supplement: Supplementary file 1 — Supplementary Information [file 41541_2021_284_MOESM1_ESM.pdf]

Supplementary Materials

Title

A Single Dose Investigational Subunit Vaccine for Human Use against Nipah virus and Hendra virus

Running title

Single dose Nipah human vaccine efficacy

Authors

Thomas W. Geisbert,[1,2] Kathryn Bobb,[3] Viktoriya Borisevich,[1,2] Joan B. Geisbert,[1,2] Krystle N. Agans,[1,2] Robert W. Cross,[1,2] Abhishek N. Prasad,[1,2] Karla A. Fenton,[1,2] Hao Yu,[3] Timothy R. Fouts,[3] Christopher C. Broder,[4] and Antony S. Dimitrov[3,4,*]

Affiliations

[1]Galveston National Laboratory, University of Texas Medical Branch, Galveston, TX 77550, USA. [2]Department of Microbiology and Immunology, University of Texas Medical Branch, Galveston, TX 77550, USA. [3]Profectus BioSciences, Inc., Baltimore, MD 21224, USA. [4]Department of Microbiology and Immunology, Uniformed Services University, Bethesda, MD 20814, USA.

[*]To whom correspondence should be addressed: antony.dimitrov.ctr@usuhs.edu

Supplementary Figures 1 to 3

Supplementary Tables 1 to 9

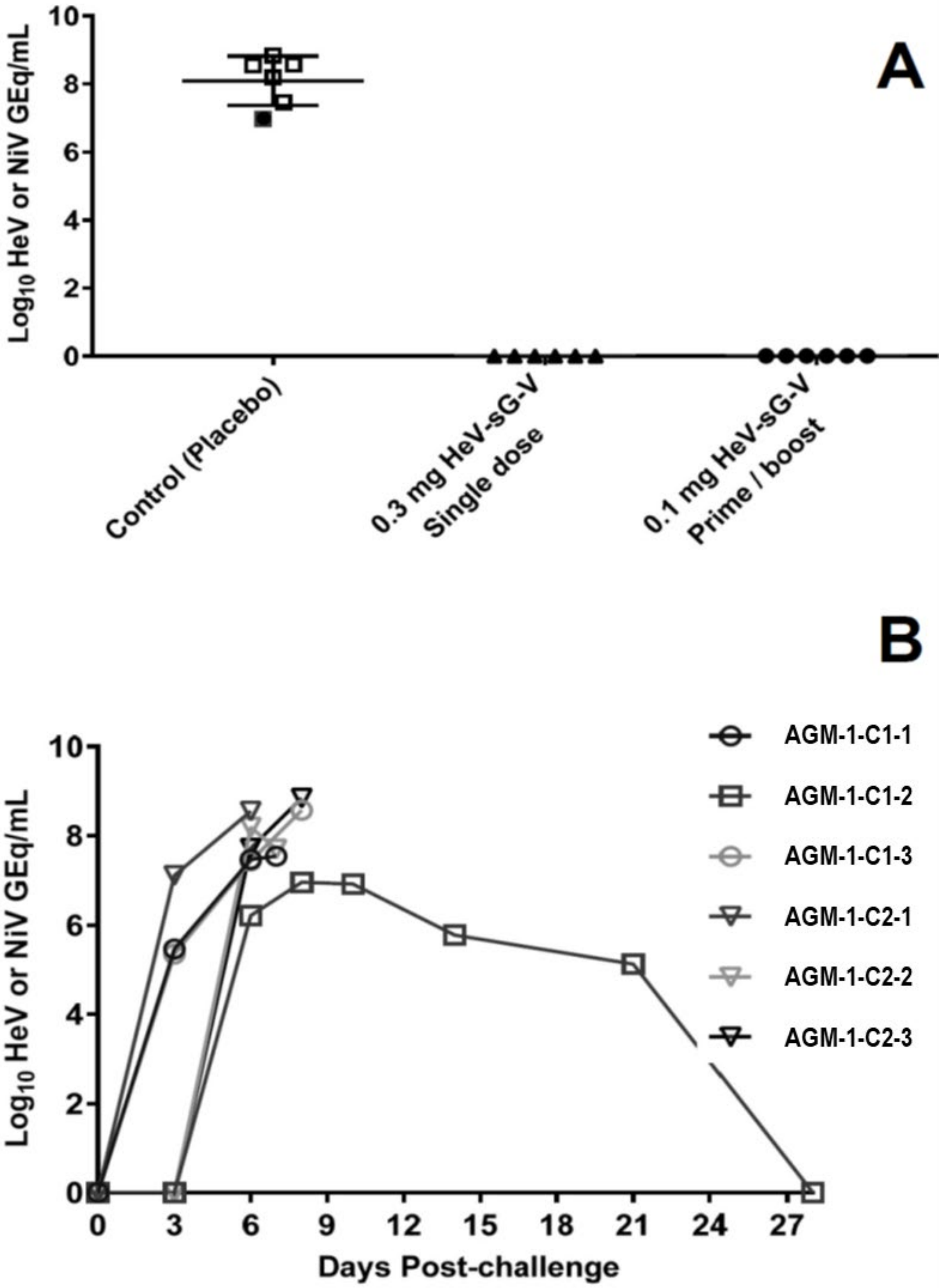

Supplementary Fig. 1. Study 1 viral RNA in LOG_{10} average genome equivalents (GEq)/mL: (**A**) Blood samples RNA peak readings for all animals, controls and immunized. The filled square (■) corresponds to the survived control peak reading. None of the vaccinated AGM showed any detectable viral RNA reading; (**B**) Blood RNA loads for control AGMs. The survived AGM-1-C1-2 was able to clear viral RNA in the blood to undetectable at the study end.

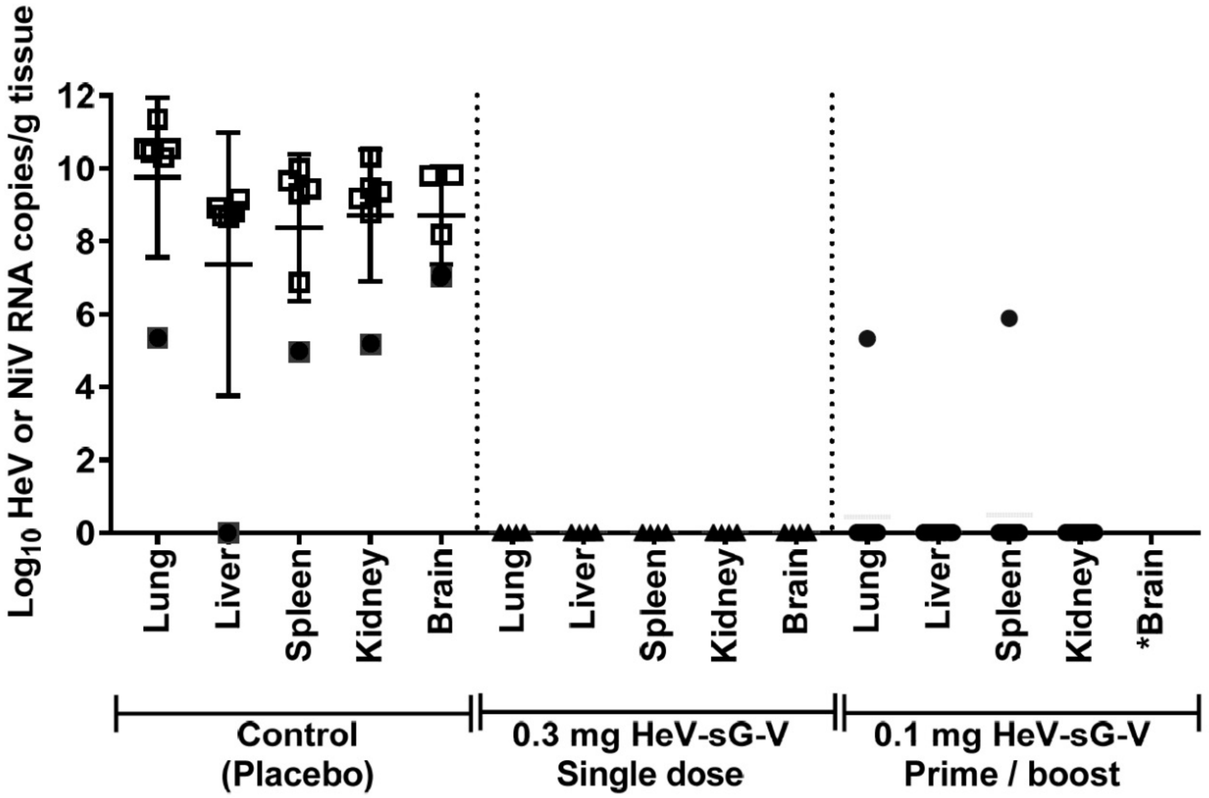

Supplementary Fig. 2. Study 1 tissue viral RNA loads in LOG_{10} average genome equivalents (GEq)/g. Viral RNA in tissue for the survived control, AGM-1-C1-2 denoted with (■), trended lower than the tissue viral load for the controls succumbed to disease. There was one AGM, 09113, denoted with (●), from the prime/boost group that had viral RNA in the lung and spleen but not in other tested tissues. All other immunized AGMs were sterilely protected.

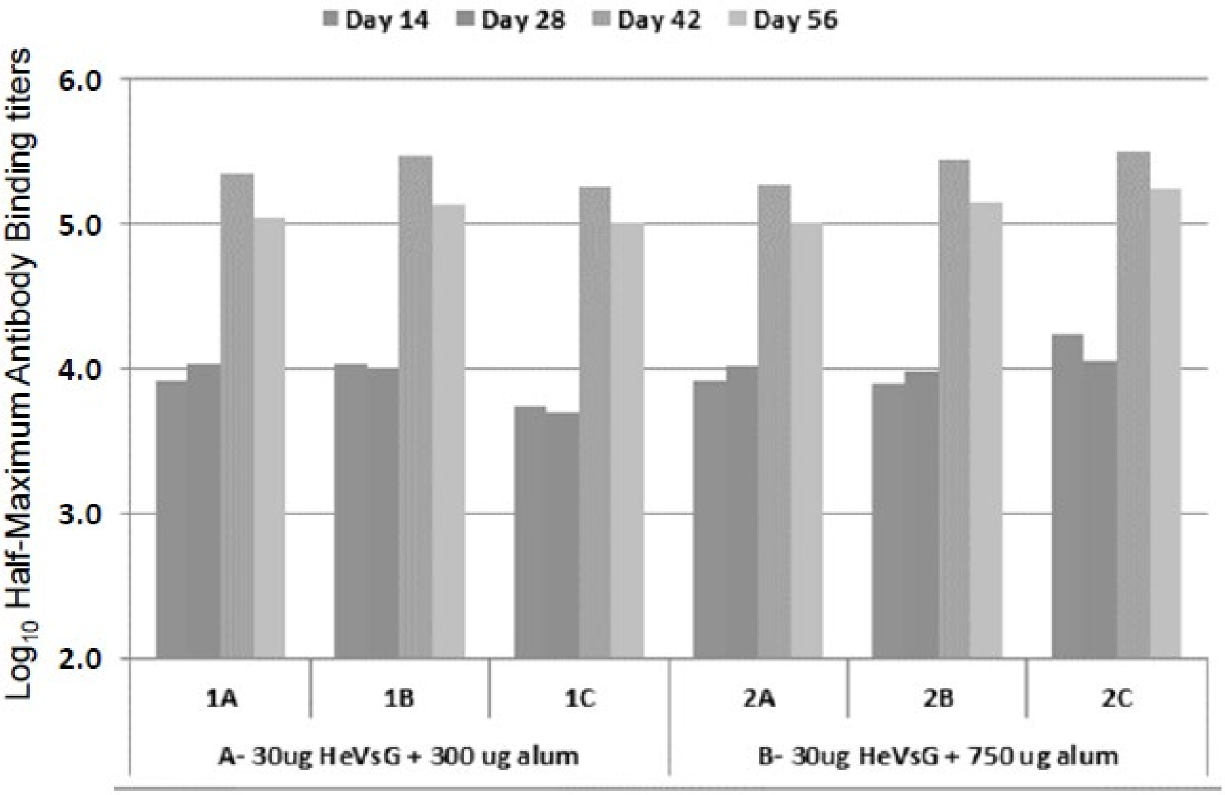

Supplementary Fig. 3. Immunogenicity study to test HeV-sG/Alum ratio effect. Two groups of three rabbits were immunized with formulation at 1/10 ratio of HeV-sG to Al^{3+} (rabbits 1A, 1B, and 1C) and at 1/25 ratio of HeV-sG to Al^{3+} (rabbits 2A, 2B, and 2C). Sera were tittered for anti-HeV-sG antibodies. Results are groupped for each rabbit – titers from left to right for days 14, 28 (the day of boost dosing), 42, and 56 (study end day). Both formulations showed indistinguishable immune responses, measured in serum titers.

NHP	Sex	Virus	Regimen	Clinical illness	Clinical pathology
AGM-1-1-1	F	HeV	Single Dose	None. Subject survived to study endpoint (d28).	Lymphocytopenia (d10, 14); granulocytopenia (d6, 8, 10, 14, 28); erythrocytopenia (d14); thrombocytopenia (d14); anemia (d14)
AGM-1-1-2	M	HeV	Single Dose	None. Subject survived to study endpoint (d28).	Granulocytopenia (d14)
AGM-1-1-3	F	HeV	Single Dose	None. Subject survived to study endpoint (d28).	Lymphocytopenia (d10); erythrocytopenia (d10); monocytopenia (d14); monocytosis (d28)
AGM-1-2-1	M	NiV-B	Single Dose	None. Subject survived to study endpoint (d28).	Monocytopenia (d28)
AGM-1-2-2	F	NiV-B	Single Dose	None. Subject survived to study endpoint (d28).	Thrombocytopenia (d6); granulocytopenia (d8, 14); erythrocytopenia (d14); anemia (d14); monocytosis (d6, 14, 28); lymphocytosis (d28); hypoglycemia (d6, 21)
AGM-1-2-3	F	NiV-B	Single Dose	None. Subject survived to study endpoint (d28).	Lymphocytopenia (d3, 6, 8, 10, 14, 28); monocytopenia (d14); erythrocytopenia (d14); anemia (d14); hypoglycemia (d3, 8)
AGM-1-3-1	M	HeV	Prime/Boost	None. Subject survived to study endpoint (d28).	Lymphocytopenia (d10); monocytopenia (d10); granulocytopenia (d14)
AGM-1-3-2	M	HeV	Prime/Boost	None. Subject survived to study endpoint (d28).	Lymphocytopenia (d6); thrombocytopenia (d3, 14); monocytosis (d3); erythrocytopenia (d14); anemia (d14)
AGM-1-3-3	F	HeV	Prime/Boost	None. Subject survived to study endpoint (d28).	Lymphocytopenia (d10); ALT ↑ (d21)
AGM-1-3-4	M	HeV	Prime/Boost	None. Subject survived to study endpoint (d28).	Lymphocytopenia (d6, 10, 14); monocytopenia (d6, 10); erythrocytopenia (d10, 14); thrombocytopenia (d14); anemia (d14); granulocytopenia (d14)
AGM-1-3-5	F	HeV	Prime/Boost	None. Subject survived to study endpoint (d28).	Lymphocytopenia (d10); monocytopenia (d6, 14); granulocytopenia (d14); erythrocytopenia (d14); anemia (d14); hypoglycemia (d8); ALT ↑ (d21)
AGM-1-3-6	F	HeV	Prime/Boost	None. Subject survived to study endpoint (d28).	Lymphocytopenia (d10); granulocytopenia (d8)
AGM-1-4-1	M	NiV-B	Prime/Boost	None. Subject survived to study endpoint (d28).	Monocytopenia (d6)
AGM-1-4-2	M	NiV-B	Prime/Boost	None. Subject survived to study endpoint (d28).	Granulocytopenia (d3, 14, 28); monocytosis (d3, 6, 8, 14, 21, 28); hypoglycemia (d28)

AGM-1-4-3	M	NiV-B	Prime/Boost	None. Subject survived to study endpoint (d28).	Lymphocytopenia (d6, 14); erythrocytopenia (d14); anemia (14)
AGM-1-4-4	F	NiV-B	Prime/Boost	None. Subject survived to study endpoint (d28).	Monocytosis (d21)
AGM-1-4-5	F	NiV-B	Prime/Boost	None. Subject survived to study endpoint (d28).	Lymphocytosis (d8); monocytosis (d8, 28); granulocytosis (d6, 14, 21)
AGM-1-4-6	F	NiV-B	Prime/Boost	None. Subject survived to study endpoint (d28).	Granulocytopenia (d3); thrombocytopenia (d14); monocytopenia (d14); erythrocytopenia (d14); granulocytosis (d6); monocytosis (d21); lymphocytosis (d21)
AGM-1-C1-1	M	HeV	Control	Depression (d7); weakness (d7); recumbency (d7); abdominal breathing (d7). Subject succumbed to disease (d7).	Lymphocytopenia (d6); granulocytopenia (d3, 6); thrombocytopenia (d6, 7); monocytosis (d3, 6, 7); granulocytosis (d7); hyperglycemia (d7); CRE ↑ (d7); hypoalbuminemia (d7); hypoproteinemia (d7); CRP ↑ (d7)
AGM-1-C1-2	M	HeV	Control	Anorexia (d9, 10); tachypnea (d9-11); depression (d10). Subject survived to study endpoint (d28).	Lymphocytopenia (d6, 8); monocytopenia (d6); thrombocytopenia (d8, 10); monocytosis (d14); BUN ↑ (d10); hypoalbuminemia (d10); hypoamylasemia (d3); CRP ↑ (d8, 10)
AGM-1-C1-3	F	HeV	Control	Anorexia (d8); depression (d8); weakness (d8); hypothermia (d8); recumbency (d8); unresponsiveness (d8); bradypnea (d8). Subject succumbed to disease (d8).	Lymphocytopenia (d3, 6); monocytopenia (d6); thrombocytopenia (d8); granulocytosis (d8); hypoglycemia (d8); CRE ↑ (d8); hypoalbuminemia (d8); hypoproteinemia (d8); hypoamylasemia (d6); CRP ↑ (d8)
AGM-1-C2-1	M	NiV-B	Control	Anorexia (d6); hypothermia (d6); depression (d6); weakness (d6); recumbency (d6); unresponsiveness (d6); bradypnea (d6); nasal exudate (d6). Subject succumbed to disease (d6).	Lymphocytopenia (d3, 6); monocytosis (d6); granulocytopenia (d3); thrombocytopenia (d6); granulocytosis (d6); BUN ↑ (d6); CRE ↑ (d6); hypoalbuminemia (d6); hypoproteinemia (d6); hypoamylasemia (d6); AST ↑↑↑↑ (d6); ALP ↓ (d6)
AGM-1-C2-2	M	NiV-B	Control	Pleural effusion (d6); abdominal breathing (d7); depression (d7); weakness (d7); recumbency (d7); unresponsiveness (d7); bradypnea (d7). Subject succumbed to disease (d7).	Lymphocytopenia (d6); monocytopenia (d7); granulocytopenia (d3, 6); thrombocytopenia (d7); BUN ↑ (d7);); CRE ↑ (d7); hypoalbuminemia (d7); AST ↑ (d7); CRP ↑ (d6); ↑↑↑ (d7)
AGM-1-C2-3	F	NiV-B	Control	Anorexia (d8); depression (d8); weakness (d8); hypothermia (d8); recumbency (d8); unresponsiveness (d8); abdominal breathing (d8). Subject succumbed to disease (d8).	Lymphocytopenia (d8); thrombocytopenia (d8); monocytosis (d6, 8); granulocytosis (d8); hypoglycemia (d3); hyperglycemia (d8); BUN ↑ (d8); CRE ↑ (d8); hypoalbuminemia (d8); CRP ↑ (d8)

Supplementary Table 1. Clinical description and outcome of HeV-sG-vaccinated African green monkeys following henipavirus challenge. Days after henipavirus challenge are in parentheses. All reported findings are in comparison to baseline (d0) values. Anorexia is defined as no food consumed from the previous day. Fever is defined as a temperature more than 2.5 °F over baseline, or at least 1.5 °F over baseline and ≥ 103.5 °F. Hypothermia is defined as a temperature ≤3.5°F below baseline. Lymphocytopenia, monocytopenia, erythrocytopenia, thrombocytopenia, and granulocytopenia are defined by a ≥35% drop in numbers of lymphocytes, monocytes, erythrocytes, platelets, or granulocytes, respectively. Lymphocytosis, monocytosis, and granulocytosis are defined by a 100% or greater increase in numbers of lymphocytes, monocytes, or granulocytes, respectively. Hyperglycemia is defined as a 100% or greater increase in levels of glucose. Hypoglycemia is defined by a ≥25% decrease in levels of glucose. Anemia is defined as a concurrent ≥25% decrease in erythrocyte count, Hct, and Hgb. Hypoalbuminemia is defined by a ≥25% decrease in levels of albumin. Hypoproteinemia is defined by a ≥25% decrease in levels of total protein. Hypoamylasemia is defined by a ≥25% decrease in levels of serum amylase. Hyperamylasemia is defined as a ≥ 100% increase in levels of serum amylase. Hypocalcemia is defined by a ≥25% decrease in levels of serum calcium. Increases in ALT, AST, ALP, CRE, CRP, Hct, and Hgb were graded on the following scale: ↑ = 1-5 fold (2-5 fold for ALT/AST), ↑↑ = >5-10 fold, ↑↑↑ = >10-20 fold, ↑↑↑↑ = >20-fold, ↓ = ≥50% decrease. (BUN) blood urea nitrogen, (ALT) alanine aminotransferase, (AST) aspartate aminotransferase, (ALP) alkaline phosphatase, (CRE) Creatinine, (CRP) C-reactive protein, (Hct) hematocrit, (Hgb) hemoglobin.

NHP	Sex	Regimen	Clinical illness	Clinical pathology
AGM-2-1-1	F	100 µg, 7dbc	Anorexia (d5). Subject survived to study endpoint (d28).	Monocytopenia (d4, 21, 28); granulocytopenia (d4, 7, 28); lymphocytosis (d10, 14)
AGM-2-1-2	M	100 µg, 7dbc	None. Subject survived to study endpoint (d28).	Monocytopenia (d4, 7, 21); granulocytopenia (d4, 7); lymphocytosis (d14); monocytosis (d10, 14)
AGM-2-1-3	M	100 µg, 7dbc	Anorexia (d9); abdominal breathing (d9, 10); open-mouth breathing (d9); rhinorrhea (d9); tachypnea (d11, 12); hypothermia (d21). Subject survived to study endpoint (d28).	Granulocytopenia (d4, 10, 28); thrombocytopenia (d10); lymphocytosis (d14, 21); monocytosis (d7, 10, 14, 21, 28); CRP ↑ (d7, 10)
AGM-2-2-1	F	100 µg, 14dbc	Abdominal breathing (d8). Subject survived to study endpoint (d28).	Granulocytopenia (d4, 10, 21, 28); monocytosis (d7, 10, 14, 21, 28); hypoglycemia (d10)
AGM-2-2-2	F	100 µg, 14dbc	None. Subject survived to study endpoint (d28).	Monocytopenia (d4, 10, 14, 21, 28); granulocytopenia (d4, 14, 21, 28)
AGM-2-2-3	M	100 µg, 14dbc	None. Subject survived to study endpoint (d28).	Thrombocytopenia (d10); lymphocytosis (d10); monocytosis (d10)
AGM-2-2-4	M	100 µg, 14dbc	None. Subject survived to study endpoint (d28).	Granulocytopenia (d4, 7); thrombocytopenia (d7); monocytosis (d7, 10, 14); lymphocytosis (d10, 14, 28)
AGM-2-3-1	F	300 µg, 14dbc	Anorexia (d6, 20, 21, 24, 25); depression (d20-25); weakness (d20-25); ptosis (d20-25); facial palsy (d20-23); hunched posture (d22-24); tremor (d22, 24); recumbency (d25); bradypnea (d25); hypothermia (d21, 25). Subject succumbed to disease (d25).	Monocytosis (d4, 7, 10, 14); lymphocytopenia (d25); monocytopenia (d25); granulocytopenia (d4, 7, 25); granulocytosis (d10); hyperglycemia (d21, 25); BUN ↑ (d21), ↑↑ (d25); CRE ↑↑ (d25); hyperamylasemia (d25); ALP ↑ (d25); CRP ↑↑↑ (d25)
AGM-2-3-2	F	300 µg, 14dbc	None. Subject survived to study endpoint (d28).	Monocytopenia (d4); granulocytopenia (d7)
AGM-2-3-3	M	300 µg, 14dbc	None. Subject survived to study endpoint (d28).	Granulocytopenia (d10); monocytosis (d14)
AGM-2-3-4	M	300 µg, 14dbc	None. Subject survived to study endpoint (d28).	Monocytopenia (d7); granulocytosis (d14); CRE ↑ (d28); CRP ↑ (d7)
AGM-2-C-1	M	Control	Fever (d7); abdominal breathing (d7). Subject succumbed to disease (d8).	Lymphocytopenia (d7); granulocytopenia (d4); monocytosis (d7); granulocytosis (d7); hypoglycemia (d7); hypoamylasemia (d7); CRP ↑ (d7)
AGM-2-C-2	F	Control	Anorexia (d6); depression (d6); weakness (d6); hunched posture (d6); hypothermia (d6); recumbency (d6); tachypnea (d6); abdominal breathing (d6). Subject succumbed to disease (d6).	Thrombocytopenia (d6); monocytosis (d6); granulocytosis (d6); BUN ↑ (d6); CRE ↑ (d6); hypoalbuminemia (d6); hypoproteinemia (d6); hyperamylasemia (d6); CRP ↑ (d6)

Supplementary Table 2. Clinical description and outcome of HeV-sG-vaccinated African green monkeys following NiV-B challenge. Abreviation "dbc" stands for "days before challenge". Days after NiV-B challenge are in parentheses. All reported findings are in comparison to baseline (d0) values. Anorexia is defined as no food consumed from the previous day. Fever is defined as a temperature more than 2.5 °F over baseline, or at least 1.5 °F over baseline and ≥ 103.5 °F. Hypothermia is defined as a temperature ≤3.5°F below baseline. Lymphocytopenia, monocytopenia, erythrocytopenia, thrombocytopenia, and granulocytopenia are defined by a ≥35% drop in numbers of lymphocytes, monocytes, erythrocytes, platelets, or granulocytes, respectively. Lymphocytosis, monocytosis, and granulocytosis are defined by a 100% or greater increase in numbers of lymphocytes, monocytes, or granulocytes, respectively. Hyperglycemia is defined as a 100% or greater increase in levels of glucose. Hypoglycemia is defined by a ≥25% decrease in levels of glucose. Anemia is defined as a concurrent ≥25% decrease in erythrocyte count, Hct, and Hgb. Hypoalbuminemia is defined by a ≥25% decrease in levels of albumin. Hypoproteinemia is defined by a ≥25% decrease in levels of total protein. Hypoamylasemia is defined by a ≥25% decrease in levels of serum amylase. Hyperamylasemia is defined as a ≥ 100% increase in levels of serum amylase. Hypocalcemia is defined by a ≥25% decrease in levels of serum calcium. Increases in ALT, AST, ALP, CRE, CRP, Hct, and Hgb were graded on the following scale: ↑ = 1-5 fold (2-5 fold for ALT/AST), ↑↑ = >5-10 fold, ↑↑↑ = >10-20 fold, ↑↑↑↑ = >20-fold, ↓ = ≥50% decrease. (BUN) blood urea nitrogen, (ALT) alanine aminotransferase, (AST) aspartate aminotransferase, (ALP) alkaline phosphatase, (CRE) Creatinine, (CRP) C-reactive protein, (Hct) hematocrit, (Hgb) hemoglobin.

AGM /Group	Day	RNA copies (GEq/ml)	LOG_{10} RNA copies / ml
AGM-2-C-1 / Control	D0	<LOD	<LOD
	D7	$1.24x10^7$	7.09
AGM-2-C-2 / Control	D0	<LOD	<LOD
	D4	$6.04x10^5$	5.78
	D6	$5.60x10^7$	7.75
AGM-2-1-1 / Group 1	D4	$1.54x10^5$	5.19
	D7	$2.88x10^5$	5.46
AGM-2-1-2 / Group 1	D7	$1.34x10^6$	6.13
AGM-2-1-3 / Group 1	D7	$4.74x10^6$	6.68
	D10	$3.33x10^6$	6.52

Supplementary Table 3. Viral RNA in blood samples in Study 2 measured by qRT-PCR. All data for samples with RNA copy reading bellow LOD (Limit of Detection, $1.00x10^3$ GEq/mL) are skipped and only data for samples with positive readings are shown in the table. Data for both controls AGM-2-C-1 and AGM-2-C-2 blood samples at the day of challenge, D0, are shown for reference.

Tissue sample from:	LOG10 (RNA copies (GEq/gram))					
	AGM-2-C-2 Control	AGM-2-C-1 Control	AGM-2-1-2 Group 1	AGM-2-1-1 Group 1	AGM-2-3-4 Group 3	AGM-2-3-1 Group 3
Axillary LN	7.91	7.58	5.22			
Inguinal LN	8.02	7.96	5.36			
Liver	7.85	7.01			5.31	
Spleen	5.45	8.16	5.59	5.75	6.30	
Kidney	7.91	7.83	5.04			
Adrenal	7.68	7.26				
Lung RU	9.72	8.57				
Lung RM	9.66	9.34				
Lung RL	9.41	9.13				
Lung LU	9.74	9.02				
Lung LM	9.29	9.30				
Lung LL	9.54	9.28				
Brain Front	6.69	6.95				
Brain Stem	7.60	7.60			5.23	6.12
CSC	6.80	5.77	4.85			4.92
Pancreas	6.44	6.44				
Urinary bladder	6.30	6.98				
Gonad	7.71	6.83				
Uterus or Prostate	7.60	7.41				
Conjunctiva	6.76	7.36				
Eye	6.30	6.96				

Supplementary Table 4. Study 2 tissue samples viral RNA measured by qRT-PCR. Only samples with RNA copy reading above LOD (Limit of Detection, $1.00x10^3$ GEq/gram) are shown. Missing number in the table means measured value is below LOD. The AGM that died on D25 had NiV RNA in brain stem and CSC, however, no virus could be isolated from these tissues.

	AGM ID	Challenge Day	End of Study Day
Control	AGM-2-C-2	< LOD	n/a
	AGM-2-C-1	< LOD	n/a
Group 1	AGM-2-1-1	< LOD	1280
	AGM-2-1-2	< LOD	160
	AGM-2-1-3	< LOD	640
Group 2	AGM-2-2-1	20	320
	AGM-2-2-2	10	320
	AGM-2-2-3	< LOD	320
	AGM-2-2-4	< LOD	320
Group 3	AGM-2-3-1	< LOD	320*
	AGM-2-3-2	< LOD	160
	AGM-2-3-3	10	160
	AGM-2-3-4	< LOD	640

Supplementary Table 5. Study 2 Serum Neutralization Titers (SNT) from samples at the challenge day and termination day. Asterisk (*) refers to the AGM that died on D25, which day was the end of study day for this animal. The limit of detection (LOD) for this assay is 10.

ID number	Gender	Body weight (kg)	Treatment Group
AGM-1-C1-1	M	5.17	Control, HeV (C1).
AGM-1-C1-2	M	5.50	
AGM-1-C1-3	F	4.32	
AGM-1-C2-1	M	5.24	Control, NiV (C2).
AGM-1-C2-2	M	5.68	
AGM-1-C2-3	F	4.68	
AGM-1-1-1	M	3.56	Single dose, HeV (1).
AGM-1-1-2	M	4.67	
AGM-1-1-3	F	3.62	
AGM-1-2-1	M	4.62	Single dose, NiV (2).
AGM-1-2-2	F	3.52	
AGM-1-2-3	F	3.90	
AGM-1-3-1	F	4.34	Prime/Boost, HeV (3).
AGM-1-3-2	M	4.25	
AGM-1-3-3	F	3.38	
AGM-1-3-4	M	5.00	
AGM-1-3-5	F	3.70	
AGM-1-3-6	F	3.70	
AGM-1-4-1	M	4.56	Prime/Boost, NiV (4).
AGM-1-4-2	M	3.94	
AGM-1-4-3	M	5.14	
AGM-1-4-4	F	3.44	
AGM-1-4-5	F	3.68	
AGM-1-4-6	F	3.68	

Supplementary Table 6. Animal Identification Number and Group Assignment in Study 1. Body weights measured at the initial physical exam are shown in this table.

ID number	Gender	Body weight (kg)	Treatment Group
AGM-2-1-1	F	4.00	Group 1
AGM-2-1-2	M	5.12	100-mg dose
AGM-2-1-3	M	5.45	challenge on day 7 post immunization
AGM-2-2-1	F	3.44	Group 2
AGM-2-2-2	F	3.76	100-mg dose
AGM-2-2-3	M	5.54	challenge on day 14
AGM-2-2-4	M	5.09	post immunization
AGM-2-3-1	F	4.04	Group 3
AGM-2-3-2	F	3.16	300-mg dose
AGM-2-3-3	M	4.66	challenge on day 14
AGM-2-3-4	M	5.92	post immunization.
AGM-2-C-1	M	5.81	Control challenged day 14
AGM-2-C-2	F	3.30	Control challenged day 7 post immunization

Supplementary Table 7. Animal Identification Number and Group Assignment in Study 2. Body weights measured at the initial physical exam are shown in this table.

Study Day	Post Challenge Day	Whole Blood		Sera	
		Hematology	Viral RNA qRT-PCR	Serum Chemistry	Anti-HeV-sG Antibodies / SNT
0		X	X		- / X
28		X	X		- / X
56	0	X	X	X	X / X
59	3	X	X	X	X / -
62	6	X	X	X	X / -
64	8	X	X	X	X / -
66	10	X	X	X	X / -
70	14	X	X	X	X / -
77	21	X	X	X	X / -
84	28	X	X	X	X / X

Supplementary Table 8. Schedule for clinical and analytical evaluation samples collection schedule in Study 1. Quantification for antibody binding titers and serum neutralization titers (SNT) is shown in **Figs. 2** and **3**.

Study Day	Post Challenge Day	Whole Blood		Sera	
		Hematology	Viral RNA qRT-PCR	Serum Chemistry	Neutralization Titers (50%)
0	-14	X	X	X	X[#]
7	-7	X	X	X	X[#]
14	0	X	X	X	X
18	4	X	X	X	
20	6	X	X	X	
21	7	X	X	X	
24	10	X	X	X	
28	14	X	X	X	
35	21	X	X	X	
39	25	X	X	X	X
42	28	X	X	X	X

Supplementary Table 9. Schedule for clinical and analytical evaluation samples collection in Study 2. The superscript # denotes control samples taken to measure base pre-immunity (days -7 and -14). AGMs from groups 2 and 3 were sampled at day -14, but not at day -7.
