# Supplementary material for: A single dose investigational subunit vaccine for human use against Nipah virus and Hendra virus
Source: NPJ Vaccines. 2021 Feb 8;6:23. doi: 10.1038/s41541-021-00284-w (PMC7870971; doi:10.1038/s41541-021-00284-w)
Supplement: Supplementary file 2 — Reporting Summary [file 41541_2021_284_MOESM2_ESM.pdf]

nature research

Corresponding author(s): Antony S. Dimitrov
DBBR
NPJVACCINES-00627

Reporting Summary

Nature Research wishes to improve the reproducibility of the work that we publish. This form provides structure for consistency and transparency in reporting. For further information on Nature Research policies, see Authors & Referees and the Editorial Policy Checklist.

Statistical parameters

When statistical analyses are reported, confirm that the following items are present in the relevant location (e.g. figure legend, table legend, main text, or Methods section).

n/a	Confirmed	
☐	☒	The exact sample size (*n*) for each experimental group/condition, given as a discrete number and unit of measurement
☒	☐	An indication of whether measurements were taken from distinct samples or whether the same sample was measured repeatedly
☒	☐	The statistical test(s) used AND whether they are one- or two-sided<br>*Only common tests should be described solely by name; describe more complex techniques in the Methods section.*
☒	☐	A description of all covariates tested
☒	☐	A description of any assumptions or corrections, such as tests of normality and adjustment for multiple comparisons
☒	☐	A full description of the statistics including central tendency (e.g. means) or other basic estimates (e.g. regression coefficient) AND variation (e.g. standard deviation) or associated estimates of uncertainty (e.g. confidence intervals)
☒	☐	For null hypothesis testing, the test statistic (e.g. *F*, *t*, *r*) with confidence intervals, effect sizes, degrees of freedom and *P* value noted<br>*Give P values as exact values whenever suitable.*
☒	☐	For Bayesian analysis, information on the choice of priors and Markov chain Monte Carlo settings
☒	☐	For hierarchical and complex designs, identification of the appropriate level for tests and full reporting of outcomes
☒	☐	Estimates of effect sizes (e.g. Cohen's *d*, Pearson's *r*), indicating how they were calculated
☐	☒	Clearly defined error bars<br>*State explicitly what error bars represent (e.g. SD, SE, CI)*

Our web collection on statistics for biologists may be useful.

Software and code

Policy information about availability of computer code

Data collection	SoftMax Pro 5.4 microplate data analysis software (Molecular Devices)
Data analysis	SoftMax Pro 5.4 microplate data analysis software (Molecular Devices)

For manuscripts utilizing custom algorithms or software that are central to the research but not yet described in published literature, software must be made available to editors/reviewers upon request. We strongly encourage code deposition in a community repository (e.g. GitHub). See the Nature Research guidelines for submitting code & software for further information.

Data

Policy information about availability of data

All manuscripts must include a data availability statement. This statement should provide the following information, where applicable:

- Accession codes, unique identifiers, or web links for publicly available datasets
- A list of figures that have associated raw data
- A description of any restrictions on data availability

All substantial data is available in the text and the supplementary figures and tables. Details are available upon request. Materials generated will be available after appropriate material transfer agreement.

Field-specific reporting

Please select the best fit for your research. If you are not sure, read the appropriate sections before making your selection.

☒ Life sciences ☐ Behavioural & social sciences ☐ Ecological, evolutionary & environmental sciences

For a reference copy of the document with all sections, see nature.com/authors/policies/ReportingSummary-flat.pdf

Life sciences study design

All studies must disclose on these points even when the disclosure is negative.

Sample size	Animals group size was chosen based on previous results on survival after a lethal henipavirus challenge. A minimal group size was chosen allowing for a per group single exclusion.
Data exclusions	We had a single exclusion of a pregnant animal. Increased immune tolerance is believed to be a major contributing factor to an increased susceptibility and severity of infections during pregnancy.
Replication	Immunized subjects uniformly survived the lethal HeV and NiV challenged, while non-immunized subjects succumbed to the infection with only one exception -- each subject in a group can be regarded as a replicate.
Randomization	Equal numbers of male and female monkeys were used when possible.
Blinding	The study did not require blinding -- we had one product to test and the difference between treated and non-treated subjects is life or death. Blinding of the dose and time of challenge post-immunization was cost-prohibitive at BSL4.

Reporting for specific materials, systems and methods

Materials & experimental systems

n/a	Involved in the study
☒	☐ Unique biological materials
☐	☒ Antibodies
☐	☒ Eukaryotic cell lines
☒	☐ Palaeontology
☐	☒ Animals and other organisms
☒	☐ Human research participants

Methods

n/a	Involved in the study
☒	☐ ChIP-seq
☒	☐ Flow cytometry
☒	☐ MRI-based neuroimaging

Antibodies

Antibodies used	HRP-conjugated goat anti-monkey IgG secondary antibody, Kirkegaard and Perry, Inc. cat. 074-11-021<br>m102.4 recombinant human anti-HeV-sG, Lot# 50LSUB-3436-275001 manufactured by Catalent Pharma Solutions for Profectus BioSciences (used as control)<br>Monkey sera, polyclonal antibodies elicited post immunization, subject in the studies<br>anti-Nipah immunoreactivity was detected using an anti-Nipah N protein rabbit 345 primary antibody (gift from Dr. Linfa Wang to Dr. Broder)<br>biotinylated goat anti-rabbit IgG (Vector Labs #BA-1000)
Validation	Specificity of the primary antibodies was confirmed by including positive and negative controls.

Eukaryotic cell lines

Policy information about cell lines

Cell line source(s)	Hendra sol G Master Cell Bank, Lot #540758-MCB2, prepared by CRL for Profectus BioSciences, Inc., from parent HEK-293F cells, Invitrogen (Life Technologies) (cat# R79007. P/N 51-0029, lot# 1185030)
Authentication	Cells were no further authenticated
Mycoplasma contamination	COA for the MCB from Charles River Labs (CRL) includes a GP-V611.20 test showing Mycoplasma "Not Detected"

Commonly misidentified lines (See ICLAC register)	n/a

Animals and other organisms

Policy information about studies involving animals; ARRIVE guidelines recommended for reporting animal research

Laboratory animals	AGMs (Chlorocebus aethiops) from St. Kitts, purchased from PrimGen,Hines, IL
Wild animals	none
Field-collected samples	none
